# The role of SPECT/CT in painful, noninfected knees after knee arthroplasty: a systematic review and meta-analysis—a diagnostic test accuracy review

**DOI:** 10.1186/s13018-023-03687-8

**Published:** 2023-03-21

**Authors:** Luz Kelly Anzola, Nathaly Hernandez, Luis Fernando Rodriguez, Gilberto Sanguino, Ernesto Martinez, Rodrigo Lopez, Sergio Moreno, Robert Prill, Fernando Mut, Helmut Rasch, Michael Hirschmann

**Affiliations:** 1 Department of Nuclear Medicine, Clinica Reina Sofia, Bogota, Colombia; 2 Department of Nuclear Medicine, Clinica Colombia, Bogota, Colombia; 3 Fundacion Universitaria Sanitas Nuclear Medicine Postgraduate Program, Bogota, Colombia; 4 Department of Orthopedics and Traumatology, Clinica Reina Sofia, Bogota, Colombia; 5 Clinical Epidemiologist, Universidad Nacional de Colombia, Bogota, Colombia; 6 Center of Orthopaedics and Traumatology, University Hospital Brandenburg/Havel, Brandenburg Medical School Theodore Fontane, Neuruppin, Germany; 7 Department of Nuclear Medicine, Hospital Italiano, Montevideo, Uruguay; 8 Institute for Radiology and Nuclear Medicine, Kantonsspital Baselland, 4101 Bruderholz, Switzerland; 9 Department of Orthopaedic Surgery and Traumatology, Kantonsspital Baselland, 4101 Bruderholz, Switzerland

## Abstract

**Purpose:**

The aim of this systematic review was to present the current evidence on the clinical use of single-photon emission computed tomography/computed tomography (SPECT/CT) in the evaluation of noninfected painful knees after knee arthroplasty.

**Methods:**

Embase, PubMed, Google Scholar, Ovid, Scopus, Science Direct and the Cochrane Database of Systematic Reviews were searched from database inception to May 2022 following the PRISMA guidelines. As a primary outcome, we defined the role of SPECT/CT in the diagnostic approach to noninfected painful knee arthroplasty; as a secondary objective, we described the noninfection-related factors linked to painful knee arthroplasty. Pooled sensitivity, specificity, positive likelihood ratio, negative likelihood ratio, diagnostic odds ratio values and other indicators were calculated; receiver operating characteristic (ROC) curve analysis results and a summary of the areas under the curve (AUCs) from the included studies were reported. A Fagan plot, likelihood ratio plot and Deeks’ funnel plot were generated and analysed. Methodological quality was assessed using the QUADAS-2 tool, and the certainty of evidence was assessed by the GRADE approach.

**Results:**

A total of 493 publications were identified, of which eight met the inclusion criteria, with a final pooled sample size of 308 patients. The pooled sensitivity and specificity of SPECT/CT in diagnosing the source of pain in painful knee prostheses were 0.86 (95% CI: 0.75–0.93) and 0.90 (95% CI: 0.79–0.96), respectively, with pooled +LR and −LR values of 8.9 (95% CI: 4.11–19.19) and 0.15 (95% CI: 0.09–0.28). The pooled diagnostic odds ratio was 57.35, and the area under the curve was 0.94. SPECT/CT highly accurately identified different sources of pain, such as loosening of the prosthetic components, patellofemoral overloading, instability, malalignment of the components and degeneration of the patellofemoral compartment. The confidence of the estimates was moderate according to the GRADE approach.

**Conclusion:**

With demonstrated high sensitivity and specificity, as a diagnostic tool, SPECT/CT can identify the source of pain in painful knees after knee arthroplasty, particularly in cases of loosening, patellofemoral disorders and component malalignment (level of evidence III). These findings have significant clinical repercussions, such as in changing the initial diagnosis, identifying or excluding different causes of painful knee arthroplasties, guiding subsequent treatment and positively impacting the final clinical outcome. We moderately recommend the use of SPECT/CT for identifying the source of pain after knee arthroplasty according to the GRADE assessment. This review was preregistered in Prospero under code CRD42022320457.

**Supplementary Information:**

The online version contains supplementary material available at 10.1186/s13018-023-03687-8.

## Introduction

A painful knee after knee arthroplasty represents a major diagnostic challenge for orthopaedic surgeons. Despite a detailed history and a thorough clinical examination, it is often difficult to precisely identify the cause of the pain and establish a correct diagnosis [1]. Different causes have been identified as sources of pain in patients with painful knee arthroplasty, which can be categorized into intra- or extraarticular origin: aseptic loosening, malposition, infection, instability, extensor mechanical problems, and neurological or vascular disorders, hip or spine disorders and stress fractures, respectively [2]. In some patients, psychological factors such as anxiety, depression or excessive patient expectations have been identified as factors playing a role on the perception of pain before and after total knee arthroplasty [3]. To this respect, some authors have reported how depression and anxiety before total knee arthroplasty could predict poorer clinical outcome and higher pain level after surgery [4, 5]. Taking into account the multifactorial origin of painful knee arthroplasty, it becomes important to build a preoperative work-up to evaluate not only the anatomical and structural factors (detailed clinical history, physical examination, imaging tools, biomechanical approach,) but also the psychological factors known to influence the clinical outcome.

Assessment of the painful knee requires a multimodal approach, as the treatment depends largely on the underlying aetiology. Imaging is an established and important pillar for the clinical assessment and evaluation of the cause of knee pain.

Conventional X-rays (XRs) are the first line of investigation, as they are commonly available and relatively cost-effective. Gross abnormalities, such as periprosthetic fractures, subsidence of prosthetic components or severe loosening, can be easily detected; however, the detailed assessment of anatomical structures is limited. Computed tomography (CT) is useful in the evaluation of cortical bone abnormalities, providing excellent anatomical detail but a suboptimal assessment of soft tissue abnormalities and intramedullary changes. CT also allows a detailed analysis of the position of the prosthetic component with regard to standardized references [6].

Several studies have shown the diagnostic value of supplemental magnetic resonance imaging in the evaluation of symptomatic joint replacement [7, 8]; however susceptibility artefacts can affect the signal, limiting the assessment of bone and soft tissues. This condition has been partially overcome in recent years through the implementation of new acquisition techniques such as conventional fast-spin-echo (FSE) [9], and the most recent multi-acquisition variable-resonance image combination (MAVRIC) MRI sequence, which has been shown to reduce susceptibility artefacts near metallic implants, allowing the identification of early and subtle changes, such as osteolysis and bone marrow oedema, close to the bone–prosthesis interface [10]. MRI has also been shown to be helpful for the analysis of rotational component alignment [11] and for the evaluation of the tissues around the components, helping to detect ruptures of tendons or distention of the capsule. In spite of the promising results reported to date, it is important to consider that the shape of the polyethylene tibial insert in MRI varies widely depending on the implant type and manufacture design. Therefore, radiologists will need to become familiar with these variations to fully understand the MRI appearance [12]. Moreover, the knowledge about effective protocols for MRI after knee replacement and their scientific evaluation has been limited to few centres.

Planar scintigraphy (bone scanning) has a high but varying sensitivity with limited specificity for several pathologies. By performing tomographic reconstruction through SPECT, it is possible to obtain images with better contrast resolution while improving the anatomical localization and characterization of the lesions [13]. Furthermore, this modality allows the evaluation of several joints simultaneously [14].

Hybrid SPECT/CT, with a radiation burden of only 2–4 mSv, has emerged as an imaging tool with important advantages for diagnosing painful knees after arthroplasty: (a) it offers the anatomical detail and spatial resolution of CT while enhancing the specificity of SPECT [15]; (b) it can be used to detect osteoblastic activity between multiple time points and providing insights into treatment outcomes for individual patients [16]; (c) it can provide guidance for choosing treatment options in patients with postoperative knee pain [17]; (d) it can reveal stress in the subchondral bone, which correlates with the source of pain [18]; and (e) it can help detect functional changes before abnormalities can be seen by XR, CT or MRI [19]. Published evidence on the application of SPECT/CT in the diagnostic approach to painful knees after knee arthroplasty, although scarce, has increased in recent years, and the utility of SPECT/CT has been demonstrated in different scenarios where conventional imaging tools have limitations [20]. In a recent publication using SPECT/CT in patients with painful knees after knee arthroplasty, the authors demonstrated how component positioning-related pathologies accounted for the greatest proportion, followed by patella-related problems and instability, in contrast to much of the data published in recent years; they also reported a correlation between total knee arthroplasty (TKA) component positioning and pain and argued that in SPECT images, an increase in bone tracer uptake (BTU) in femoral areas and areas around the tibial stem or tip was more specific for the identification of a pathological condition in patients with painful TKA [21]. As an added condition, technological improvements in CT and nuclear medicine devices have led to higher-resolution images, permitting better qualitative and quantitative analysis of BTU with superior anatomical correlation. In this regard, it is beneficial to highlight a standardized method reported by Hirschmann et al. (Bruderholz image scheme protocol) [22] that can be used to identify typical distribution patterns in different pathological conditions of the knee [23]. A meticulous diagnostic work-up in painful knee arthroplasty is mandatory because revision surgery should only be performed if the cause of pain is identified, whereas revision surgery for unexplained pain has shown poor outcomes [24]. The more clinical and radiological information that is included during the assessment of painful TKA, the more confident the decision regarding TKA revision can be [25]. In this clinical scenario, it is important to know the performance of different imaging tools, in particular SPECT/CT, to determine their role as part of a diagnostic algorithm for patients with pain after total knee arthroplasty.

Previous studies, be they evaluations of diagnostic tests or correlation studies, have demonstrated the good diagnostic performance of SPECT/CT in evaluating painful knee replacement; however, the sample sizes have been relatively small, and thus, the robustness of the evidence is questionable. Moreover, most of the current evidence is from observational cohort studies, and few studies examining diagnostic accuracy have been published. To date, published systematic reviews related to painful arthroplasty are scarce: Barnsley et al. [26] analysed two studies that used SPECT/CT as an index test (sensitivity 0.86, specificity 0.93); Peng et al. [27] reported an analysis of sixteen papers; however, they did not separate the results for knee and hip prostheses. Verberne [28] conducted an analysis regarding the intra- and interobserver agreement of different nuclear medicine techniques in septic and aseptic symptomatic hip and knee replacements.

This systematic review was conducted with the primary aim of analysing the best written evidence regarding diagnostic tests using ^99m^Tc-phosphate SPECT/CT in the evaluation of noninfected painful knee arthroplasty. A meta-analysis on quantitative methods was also conducted where possible. Under this PICO algorithm [29], “In adults with suspected aseptic painful knee arthroplasty, a positive result in SPECT/CT, compared with intraoperative findings, histological findings and/or clinical outcome, is able to detect the origin of pain in painful knee arthroplasty”, it was hypothesized that SPECT/CT would be capable of detecting the origin of pain in patients with painful knees after knee arthroplasty and could be included in the diagnostic algorithm for such patients, resulting in a beneficial effect on the diagnosis and subsequent treatment.

## Materials and methods

### Criteria for study consideration in this review

*Types of study*: This review included full-text reports of systematic reviews, observational studies and prospective and retrospective cohort studies, the data of which could be used for analysis purposes in the category of diagnostic studies. Studies required a sample size larger than 10 cases, which would allow the possibility of constructing a 2 × 2 table.

*Types of participants*: Adult patients with painful knees after primary (partial or total) or revision knee arthroplasty who had undergone bone SPECT/CT scintigraphy as part of their diagnostic work-up.

*Type of intervention*: intravenous ^99^mTc HMDP/^99^mTc HDP/^99^mTc MDP or intraarticular ^99^mTc sulphur colloid, SPECT/CT.

*Type of study outcome*: The final diagnosis was related to aseptic painful knee arthroplasty. The gold standard in the study was either the result of an intraoperative finding, histological examination, clinical outcome or a comparable standard result.

*Studies were excluded* if the focus was on oncology or infection-related applications or if the emphasis was placed on aspects related to generators, radiochemistry, animal models, experimental reports or physics. Descriptive studies focused solely on the pattern of BTU in which the authors did not use any comparator to infer the explanation of the findings were also excluded. Small sample size studies with fewer than 10 patients were not included, as the minimum requirements for analysis in this review were not met. Reviews, case reports, conference presentations and so on were excluded. We also excluded studies for which we were unable to separate the results for knee prostheses from those for other prostheses as well as the results from different pathologies (e.g. septic from aseptic loosening).

*Years considered*: 2010–2022.

*Languages considered*: English, Spanish, Italian.

### Search strategy

This systematic review was conducted in adherence with the PRISMA guidelines [30] and the author guidelines for conducting systematic reviews and meta‐analyses [31, 32]. This systematic review was registered on Prospero with the following number: CRD42022320457.

### Retrieval strategy

SPECT/CT, SPECT, single-photon emission computed tomography/computed tomography, knee prosthesis, knee arthroplasty and aseptic loosening were used as keywords in the literature search.

The Cochrane Central Register of Controlled Trials (CENTRAL) published in the Cochrane Library and the Embase, PubMed, Google Scholar, Ovid, Scopus and Science Direct databases were all searched. The language was restricted to English, Italian and Spanish. Grey literature was searched through clinicaltrials.gov and a summary of conferences. When possible, authors were contacted for clarification. Because SPECT/CT began to be used as a hybrid imaging modality in knee arthroplasty orthopaedic practice in 2010, there was no written evidence about its use in this topic before that year; all relevant studies published from 2010 to May 2022 were identified by using Boolean operators, MeSH and keyword search terms adapted for each electronic database (See Additional file 1: Supplement 1).

### Study selection, data extraction and management

The extraction of study characteristics focused on citation, first author, year of publication, country of publication, study design and method, setting/context, population characteristics, exposure, radiotracer used and route of administration, image interpretation, reference standard, sample size and operative characteristics of the tests in terms of sensitivity (SEN) and specificity (SPE).

Data were extracted separately by the authors using a standardized extraction tool; the decision to include or exclude an article was made by consensus reading among LKA and NH (experts in the topic). The aforementioned search strategy was used to obtain the titles and abstracts of studies with potential relevance to the review. LKA and NH independently screened the titles, abstracts and full texts for eligibility. Care was taken to ensure that multiple studies reporting on the same patient population were excluded. Therefore, duplicate records were rechecked in another round of peer review. Reviewers resolved disagreements through discussion or, if needed, by adjudication of a third reviewer (LFR). Differences were resolved by consensus.

### Quality assessment of the risk of bias in the included studies

The included studies were assessed by using the Cochrane Collaboration [33] and QUADAS-2 [34] tools to evaluate risk of bias and variation in diagnostic studies. Signalling questions were asked to evaluate the risk of bias in patient selection (could the selection of patients have introduced bias?), index test (could the conduct or interpretation of the index test have introduced bias?), reference standard (could the reference standard, its conduct or interpretation have introduced bias?) and flow and timing (could the patient flow have introduced bias?), and consequently to evaluate applicability concerns. Following a review of the signalling questions included in the tool, the risk of bias was judged as low (when all the signalling questions for a domain were answered yes), high (when any signalling question was answered no) or unclear (in situations of uncertainty over key signals).

*Certainty of evidence assessment*. The quality of the evidence or confidence in the estimates of this systematic review was assessed by using GRADE guidance [35], first in comparative studies and then between-studies, separately. Six domains of certainty of evidence were evaluated: 1. limitation in study design, risk of bias in between-study comparisons (by using the QUADAS-2 tool); 2. indirectness and applicability; 3. inconsistency in between-study comparisons; 4. imprecision; 5. publication bias; and 6. upgrading for dose effect, large effects, residual plausible bias and confounding. Data were managed in the GRADE pro GDT program to generate a GRADE evidence and summary findings table. Recommendations were accordingly generated from the quality of evidence [36] (Additional file 2: Supplement 2).

### Levels of evidence

The Oxford Levels of Evidence tool, as produced by the Oxford Centre for Evidence-Based Medicine, was used to categorize methodological quality [37]. This tool is used to investigate the validity of diagnostic tests; randomized controlled trials are classified as level I, prospective cohort studies with consecutive patients with a consistently applied reference standard and blinding are classified as level II, retrospective cohort and case control studies are classified as level III, and case series are classified as level IV.

### Statistical analysis

The methodological quality and possible bias of the included studies were assessed using R 4.0.1 based on the QUADAS-2 tool. The quality of the included literature was evaluated for risk of bias (four entries for patient selection, the index test, the reference standard and flow and timing) and clinical applicability (three entries for patient selection, the index test and the reference standard).

The data were extracted from each article and classified as true or false positives or negatives, respectively; some were directly extracted, while others were calculated from the reported results. In order to perform meta-analysis of the data, following the methodology of Vlaar et al. [38], an adjustment of the 2 × 2 table was performed and the null values or with zeros in any of the cells of TP, FP, TN and FN were replaced by 1, in order to carry out the estimations of sensitivity, specificity and SROC. A table showing the TP, FP, TN and FN values of the cells used for the purpose of the meta-analysis is attached as Additional file 5: Supplementary file 5.

Statistical analysis was performed using Stata 17MP software (StataCorp, College Station, TX) to calculate individual and pooled estimates of sensitivity (SEN), specificity (SPE), the positive likelihood ratio (LRP), the negative likelihood ratio (LRN) and the diagnostic odds ratio (DOR) and its 95% confidence interval (CI). Finally, a forest map, summary receiver operating characteristic (SROC) curve, Fagan’s line diagram and likelihood ratio (LR) dot plot were generated, and the area under the SROC curve (AUC) was calculated.

The presence of a threshold effect was examined by calculating the Spearman correlation coefficient between the log of sensitivity and the log of (1—specificity). Heterogeneity due to non-threshold effects was assessed by the *Q*-test (*P* < 0.10 indicates heterogeneity among studies) and *I*^2^ test (if *I*^2^ > 50%, the heterogeneity among the studies is considered large), and then a random effects model was used to combine the effect sizes. Deeks’ funnel plot was used to determine whether there was publication bias in the included studies: the closer the angle between the regression line and the vertical axis in Deeks’ funnel plot, the less likely it is that publication bias was present; when *P* > 0.05, publication bias was considered to be absent. The stability of the results of the diagnostic studies was tested by sensitivity analysis. When the heterogeneity was large, meta-regression analysis was used to explore the sources of heterogeneity among studies, and subgroup analysis was conducted.

## Results

**Fig. 1:**
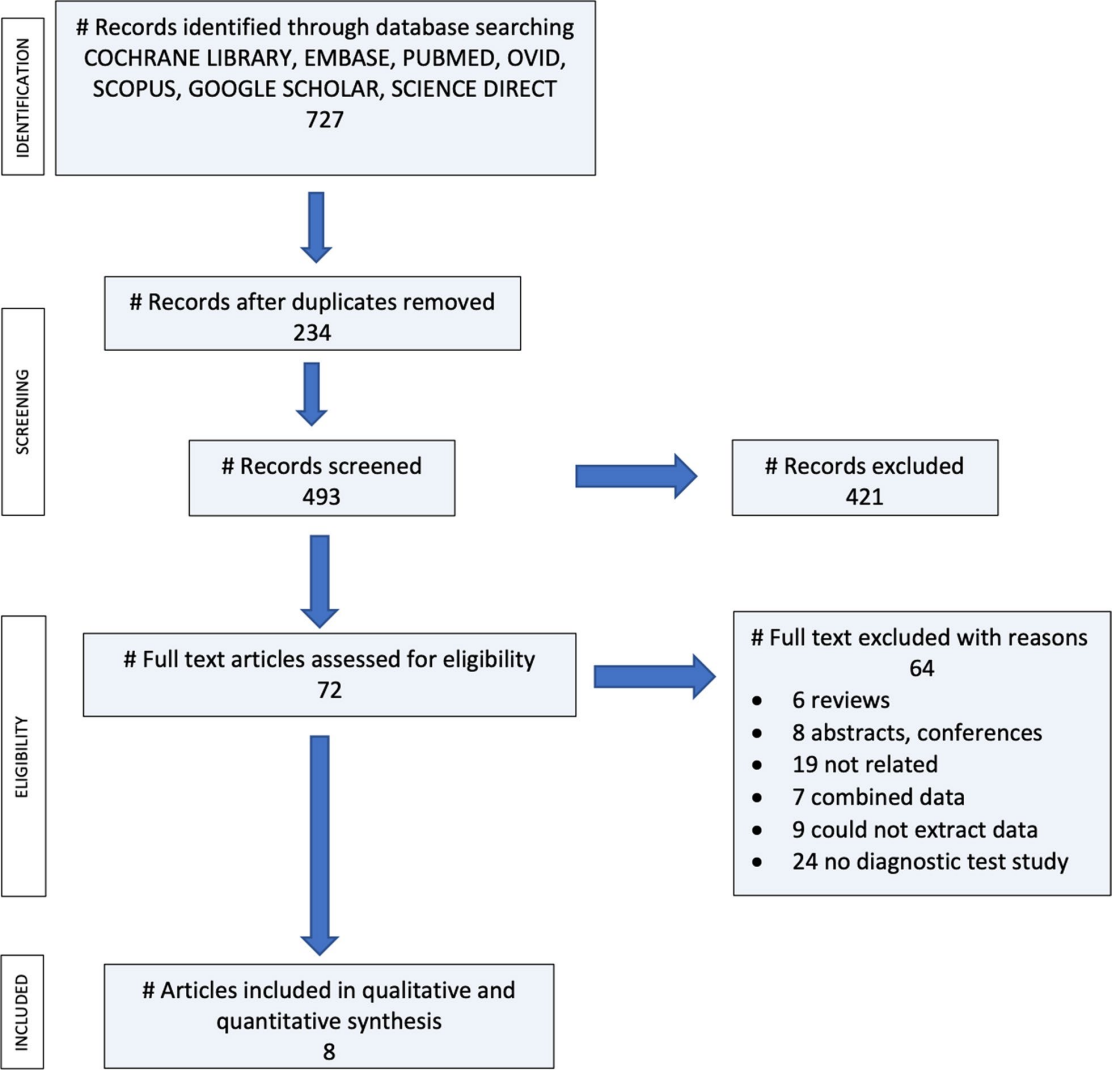
PRISMA flow chart

In this review, there were identified 727 potential studies evaluating SPECT/CT in the clinical scenario of noninfected painful knee arthroplasty. After checking for duplicates, 234 papers were immediately excluded; subsequently, 493 studies were screened, 421 were excluded (including 3 studies published in different languages), and the remaining 72 studies were assessed for eligibility. After reading the full-text records, 64 studies that did not fulfil the inclusion criteria were also excluded (8 abstracts and conferences, 6 reviews, 19 publications not related to the studied topic, 7 papers with combined data on the hips and knees that could not be separated, and 24 papers from which it was not possible to extract a 2 × 2 data table). Ultimately, a total of 8 diagnostic studies were analysed for inclusion and quantitative synthesis, as shown in Fig. 1 (PRISMA flow chart). The characteristics of the studies are summarized in Table 1.

**Table 1:** Characteristics of the included studies

Author and reference	N	Type of study	Level of evidence	Type of arthroplasty	Radiotracer	Administration	Reference test	Sensitivity	Specificity	Conclusions
Abele et al. [39]	17	Diagnostic retrospective	III	TKA	^99m^Tc Sulf Colloid	i.a	Intraoperative, follow-up	0.67	0.93	SPECT/CT arthrography with high accuracy for detecting loosening in painful knee arthroplasty
Al-Nabhani et al. [40]	24	Diagnostic retrospective	III	TKA	^99m^Tc HDP	i.v	Intraoperative, follow-up	0.90	0.80	Changed initial diagnosis in 85% of patients. SPECT/CT identified correctly loosening and excluded other causes
Arican et al. [41]	30	Diagnostic retrospective	III	TKA	^99m^Tc MDP	i.v	Intraoperative	0.87–0.93	100	Showed different SPECT/CT operative characteristics according to the anatomical place of the knee replacement. Better diagnostic performance for tibial component. A negative result excluded with high accuracy a pathological condition
Bao et al. [42]	36	Diagnostic retrospective	III	TKA	^99m^Tc Sulf Colloid	i.a	Intraoperative, follow-up	0.50	0.97	SPECT/CT arthrography showed high accuracy for detecting loosening in painful knee arthroplasty and excluding pathological condition. They showed that a detection of tracer activity along the bone interface was related to aseptic loosening
Chew et al. [43]	44	Diagnostic retrospective	III	TKA	^99m^Tc Ca Phyt	i.a	Intraoperative	0.75–0.86	0.63–0.86	They reported different diagnostic performance values according to the anatomical place of the knee replacement. Better diagnostic performance for tibial component. A negative result excluded with high accuracy a pathological condition. They demonstrated superiority of SPECT/CT when it was compared with planar images in bone scan images
Hirschmann et al. [44]	33	Diagnostic prospective	II	TKA	^99m^Tc HDP	i.v	Intraoperative	0.91	0.96	Confirmed loosening and patellofemoral disorders in all patients. They reported 1 false negative result in a patient with membrane within the interphase bone-metal. They highlighted how typical patterns of BTU significantly correlated with loosening of the components. SPECT/CT changed the clinical diagnosis in 85% of the patients
Mandegaran et al. [45]	41	Diagnostic retrospective	III	TKA	^99m^Tc MDP	i.v	Intraoperative, follow-up	0.93	0.79	SPECT/CT demonstrated additional potential aetiologies in 43% of patients: heterotopic ossification, biomechanical stress, periprosthetic fracture, degenerative patellofemoral diseases
Murer et al. [46]	83	Diagnostic retrospective	III	TKA	^99m^Tc HDP	i.v	Intraoperative	0.43–0.98	0.93–0.99	SPECT/CT changed the initial diagnosis in 65% of the patients. They provided different underlying causes of persistent knee pain after TKA and helped to identify clinically unsuspected origin of pain. They reported different values of operative characteristics according to different place of the knee replacement, highlighting the best values for tibial component and patellar structure

TKA: Total knee arthroplasty, BTU: bone tracer uptake, i.a: intraarticular, i.v: intravenous, HDP: hydroxymethylene-diphosphonate, MDP: methylene diphosphonate

According to the Oxford Levels of Evidence tool, most of the studies were considered level III.

A total of eight studies classified as diagnostic tests, with a total population of 308 patients, were analysed using the QUADAS-2 tool.

**Fig. 2:**
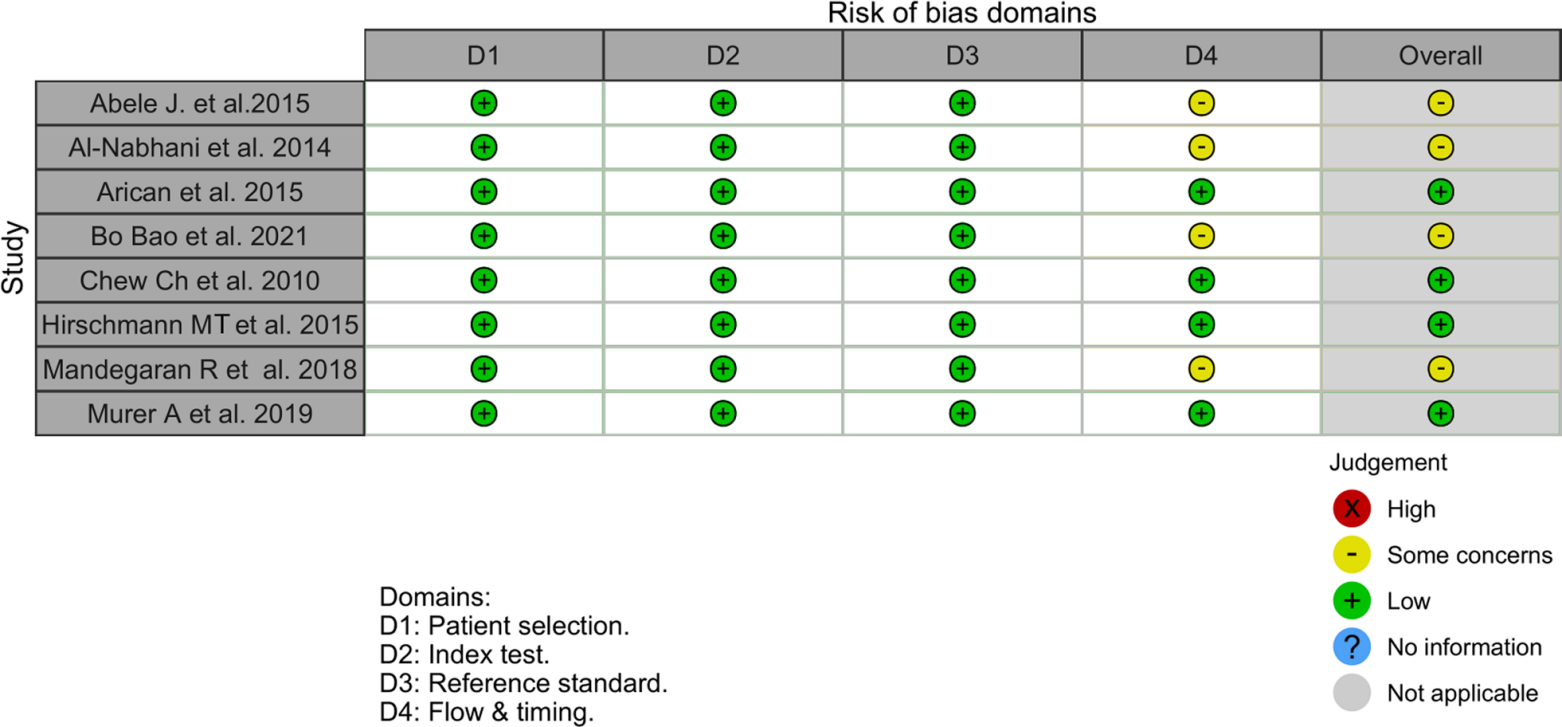
Quality assessment of the included diagnostic studies via QUADAS-2, by author

**Fig. 3:**
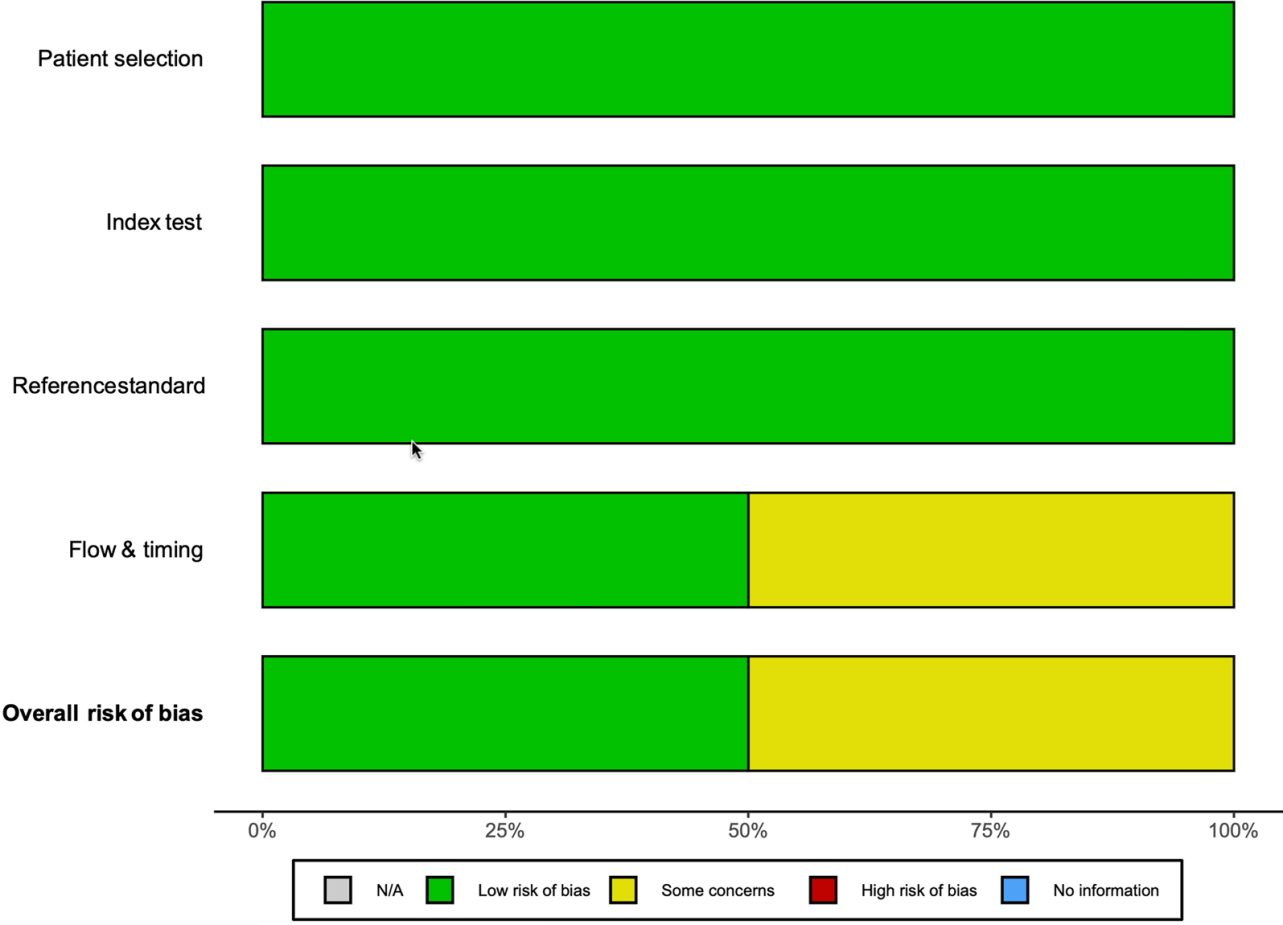
QUADAS-2 risk of bias by domains

The overall risk for the diagnostic test studies evaluated using the QUADAS-2 tool was medium. The most frequent source of bias was verification bias (flow and timing), as not all patients received the same reference standard test; the final diagnosis regarding the cause of pain was based on intraoperative findings and/or clinical follow-up assessing the response to treatment (Figs. 2 and 3).

Abele et al. [39] reported the results in a sample of 17 patients with painful knee arthroplasty and demonstrated that SPECT/CT arthrography was useful in the evaluation of suspected aseptic loosening. With a medium risk of bias under QUADAS-2 (explained by the flow and timing domain), they reported a SEN of 0.67 [95% CI: 0.09–0.99] and a SPE of 0.93 [95% CI: 0.68–1.00]. The major limitation of this study was the different reference standards that were used.

Al-Nabhani [40] reported on 24 patients with painful knee prostheses who underwent revision surgery. With a medium risk of bias under the QUADAS-2 tool (explained by the flow and timing domain), they achieved a SEN of 0.90 [95% CI: 0.55–1.00] and a SPE of 0.95 [95% CI: 0.86–0.99] for SPECT/CT in identifying the source of pain, ultimately confirming the diagnosis in 21 patients. The imaging tool confirmed mechanical loosening and excluded other causes of pain, such as infection and patellofemoral osteoarthritis.

Arican [41] reported the results for 30 patients with painful knee arthroplasties. With a low risk of bias under the QUADAS-2 tool, they showed that the performance of SPECT/CT differed according to the anatomical site of the knee replacement: for the tibial component, it achieved a SEN of 0.93 [95% CI 0.78–0.99] and a SPE of 0.0 [95% CI: 0.0–0.98]; for the femoral component, a SEN 0.87 [95% CI: 0.69–0.96] and a SPE of 0.00 [95% CI: 0.00–0.98] were obtained. However, they could not calculate an overall specificity, as all patients had undergone surgery, and there were no true-negative results on scintigraphy. They confirmed the superiority of the hybrid imaging modality over regular bone SPECT imaging. Furthermore, the authors compared the diagnostic performance of SPECT/CT between hips and knees and reported better results for the knee implants. They also demonstrated that SPECT/CT changed the initial clinical diagnosis for 16% of the patients. The major limitation of this report was the absence of true-negative patients, preventing the calculation of the overall specificity.

Bo Bao [42] reported on a retrospective cohort of 36 patients who underwent clinical and radiological evaluation for persistent pain following knee arthroplasty. With a medium risk of bias under the QUADAS-2 tool (explained by the flow and timing domain, as intraoperative findings and/or clinical or radiological evaluations were used as the reference standard), the authors reported a SEN of 0.50 [95% CI: 0.01–0.99] and a SPE of 0.97 [95% CI: 0.85–1.00] for the SPECT/CT arthrogram in detecting aseptic loosening. The authors concluded that the detection of tracer activity along the bone interface was related to aseptic loosening. The main limitation of this paper was that the surgeons were not blinded to the SPECT/CT reports, so the decision to perform the surgical revision technique used to generate the reference standard was influenced by the result of the SPECT/CT; additionally, some limitations regarding technical factors were identified that could impact the false negative results.

Chew et al. [43] reported on 44 patients who underwent SPECT/CT arthrography for the assessment of mechanical loosening of knee prosthesis. The detection of any radiotracer within the bone/prosthetic interface was interpreted as positive for loosening. With a low risk of bias under the QUADAS-2 tool, the authors showed different values for sensitivity and specificity according to the anatomical site of the knee replacement: tibial component, SEN 0.86 [95% CI: 0.42–1.00] and SPE 0.86 [95% CI: 0.71–0.95]; femoral component: SEN 0.75 [95% CI: 0.43–0.95] and SPE 0.63 [95% CI 0.44–0.79]. They also demonstrated the superiority of SPECT/CT over planar bone scan imaging. The major limitation of this study was the retrospective study design and that the surgeons were not blinded to the SPECT/CT results.

Hirschmann et al. [44] reported on 33 patients with persistent knee pain after TKA who underwent SPECT/CT as part of a routine diagnostic algorithm and for whom intraoperative findings confirmed the preoperative SPECT/CT diagnosis. With a low risk of bias under the QUADAS-2 tool, they achieved a SEN of 0.91 [95% CI: 0.59–1.00] and a SPE of 0.96 [95% CI: 0.76–1.00]. TKA loosening as well as progression of patellofemoral osteoarthritis was correctly diagnosed. They also highlighted how typical BTU patterns were significantly correlated with loosening of the components (*P* < 0.05). They observed that SPECT/CT changed the clinical diagnosis with impact at final diagnosis in 85% of patients. The major limitation of this study was that the results came from the use of only two TKA implant designs (cruciate-retaining and posterior cruciate ligament-stabilizing implants); it was not possible to generalize the results to other implant brands and designs.

Mandegaran et al. [45] obtained their results from a retrospective cohort of 41 patients who underwent SPECT/CT to identify the origin of pain among patients with painful knees after arthroplasty. With a medium risk of bias under the QUADAS-2 tool (explained by the flow and timing domain, as not all patients received the same reference standard), the reported sensitivity and specificity in identifying the source of pain in such patients were 0.93 [95% CI: 0.66–1.00] and 0.79 [95% CI: 0.59–0.92], respectively. They also reported how SPECT/CT identified an alternative diagnosis of the origin of pain in 43% of patients. They demonstrated higher diagnostic performance for SPECT/CT than ^99^mTc MDP bone scanning.

Murer et al. [46] performed a retrospective cohort study involving 83 patients with painful knees after arthroplasty who were surgically revised. With a low risk of bias under the QUADAS-2 tool, the authors showed different values for the sensitivity and specificity according to the anatomical site of the knee replacement (tibial component: SEN 0.96 [95% CI: 0.90–1.00] and SPE 0.93 [95% CI: 0.76–0.99]; femoral component: SEN 0.73 [95% CI: 0.39–0.94] and SPE 0.99 [95% CI 0.93–1.00]; patellofemoral component: SEN 0.43 [95% CI: 0.10–0.82] and SPE 0.99 [95% CI: 0.93–1.0]). With a high accuracy and a high negative predictive value, they also demonstrated how SPECT/CT changed the initial clinical diagnosis and provided different underlying causes for the persistent knee pain after TKA.

Three authors reported that SPECT/CT changed the clinical diagnosis and final treatment in 65–85% of all cases [41, 44, 46].

The best diagnostic performance with the highest sensitivity and negative predictive values was found for loosening of the prosthetic components when the bone radiotracer ^99^mTc sulphur colloid was used intraarticularly (SPECT/CT arthrography) [39, 42].

Three authors reported sensitivity and specificity values separately per compartment (tibial, femoral, patellofemoral), with the best results obtained for the tibial and patellar components [41, 43, 46].

### Meta-analysis of SPECT/CT in the diagnosis of painful knee arthroplasty

**Fig. 4:**
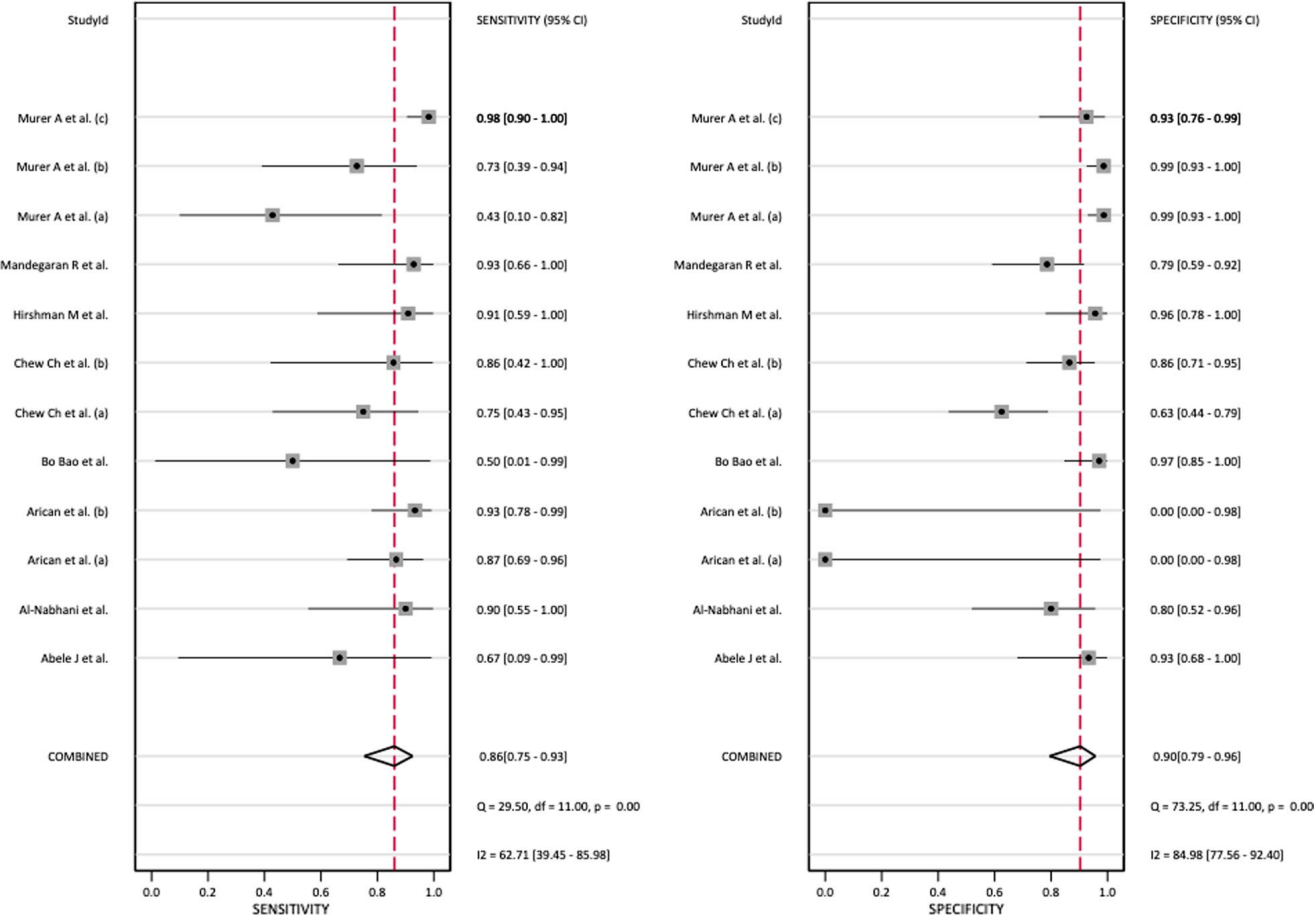
Forest plots of the sensitivities and specificities of SPECT/CT in the diagnosis of aseptic loosening. CI: confidence interval

This study used *I*^2^ in the Q test to determine whether there was a non-threshold effect for each combined effect size. The *I*^2^ value of SEN was 62.7|1 and that of SPE was 84.98 (Fig. 4 forest plot).

**Fig. 5:**
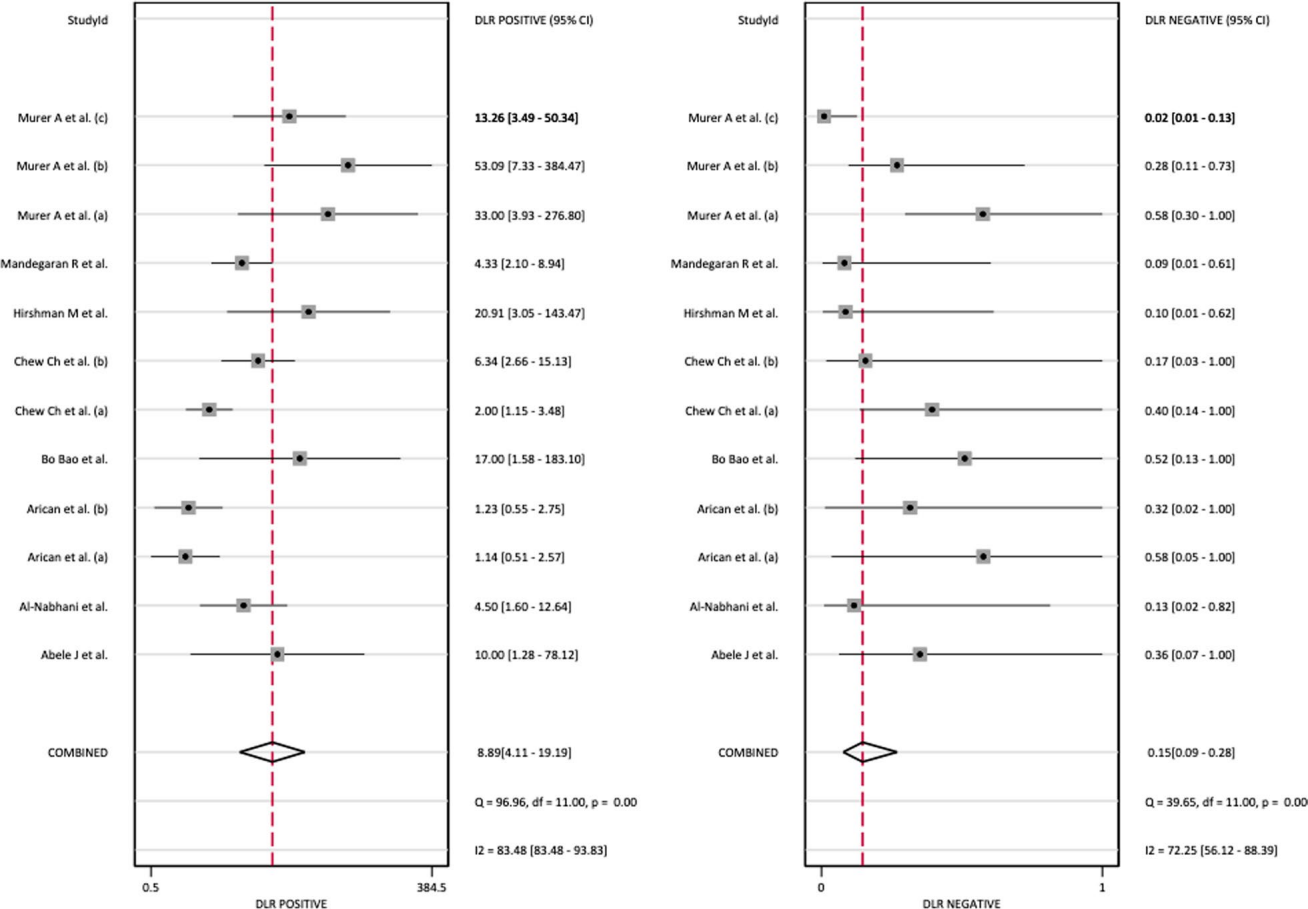
Forest plot of the combined positive diagnostic likelihood ratio and negative diagnostic likelihood ratio of SPECT/CT in the diagnosis of unhappy knee arthroplasty

**Fig. 6:**
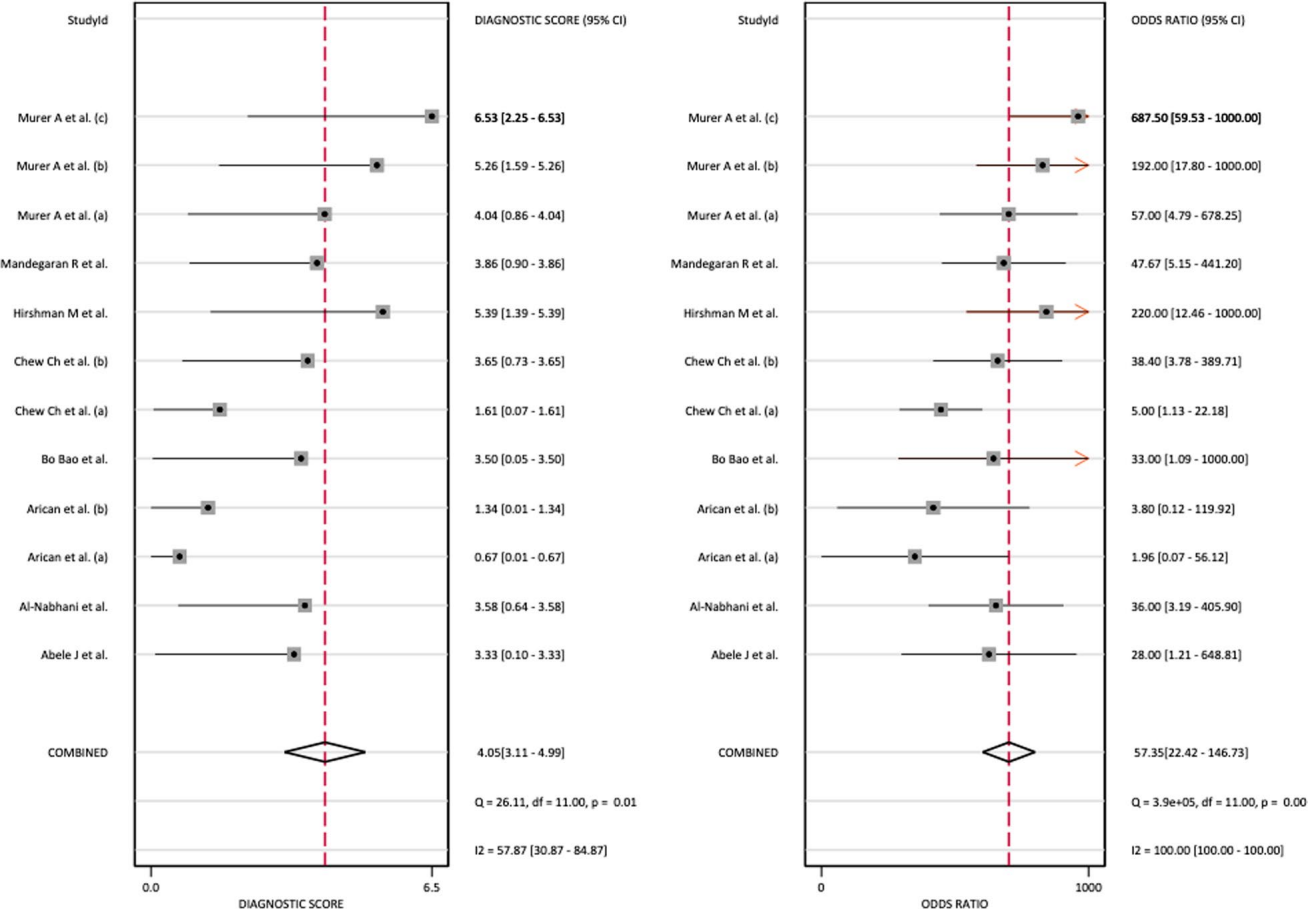
Forest plot of the combined diagnostic score and odds ratio of SPECT/CT in the diagnosis of unhappy knee arthroplasty

Because both values were greater than 50%, a bivariate normal random effects model was used for statistical analysis. The combined SEN of SPECT/CT for loosening was 0.86 (95% CI: 0.75–0.93), the combined SPE was 0.90 (95% CI: 0.79–0.96), the combined PLR was 8.89 (95% CI: 4.11–19.19), the combined NLR was 0.15 (95% CI: 0.09–0.28) (Fig. 5), and the combined DOR was 57.35 (95% CI: 25.42–146.73) (Fig. 6).

**Fig. 7:**
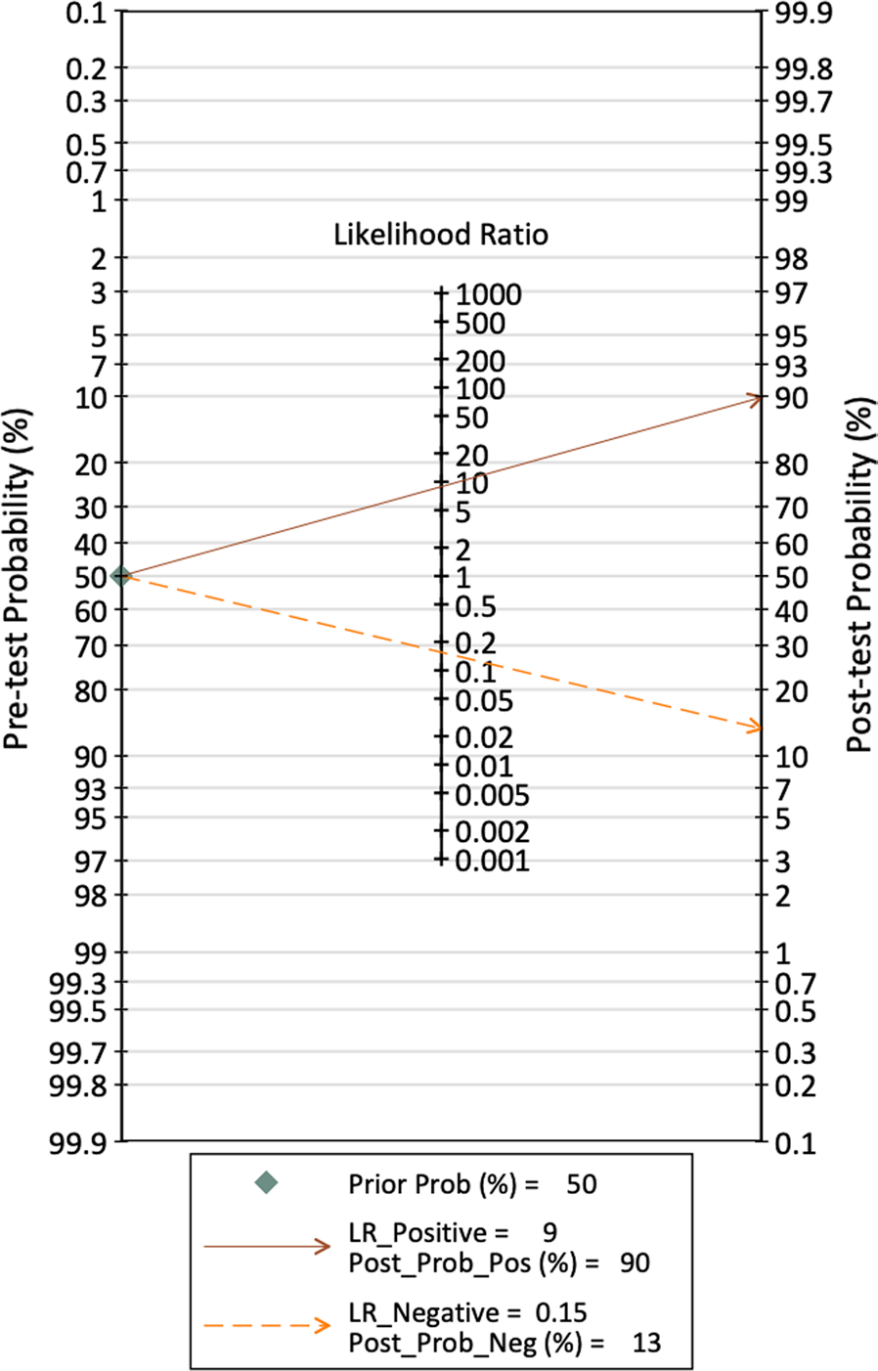
Fagan nomogram showing the clinical applicability of SPECT/CT for diagnosis. LRN: negative likelihood ratio; LRP: positive likelihood ratio

**Fig. 8:**
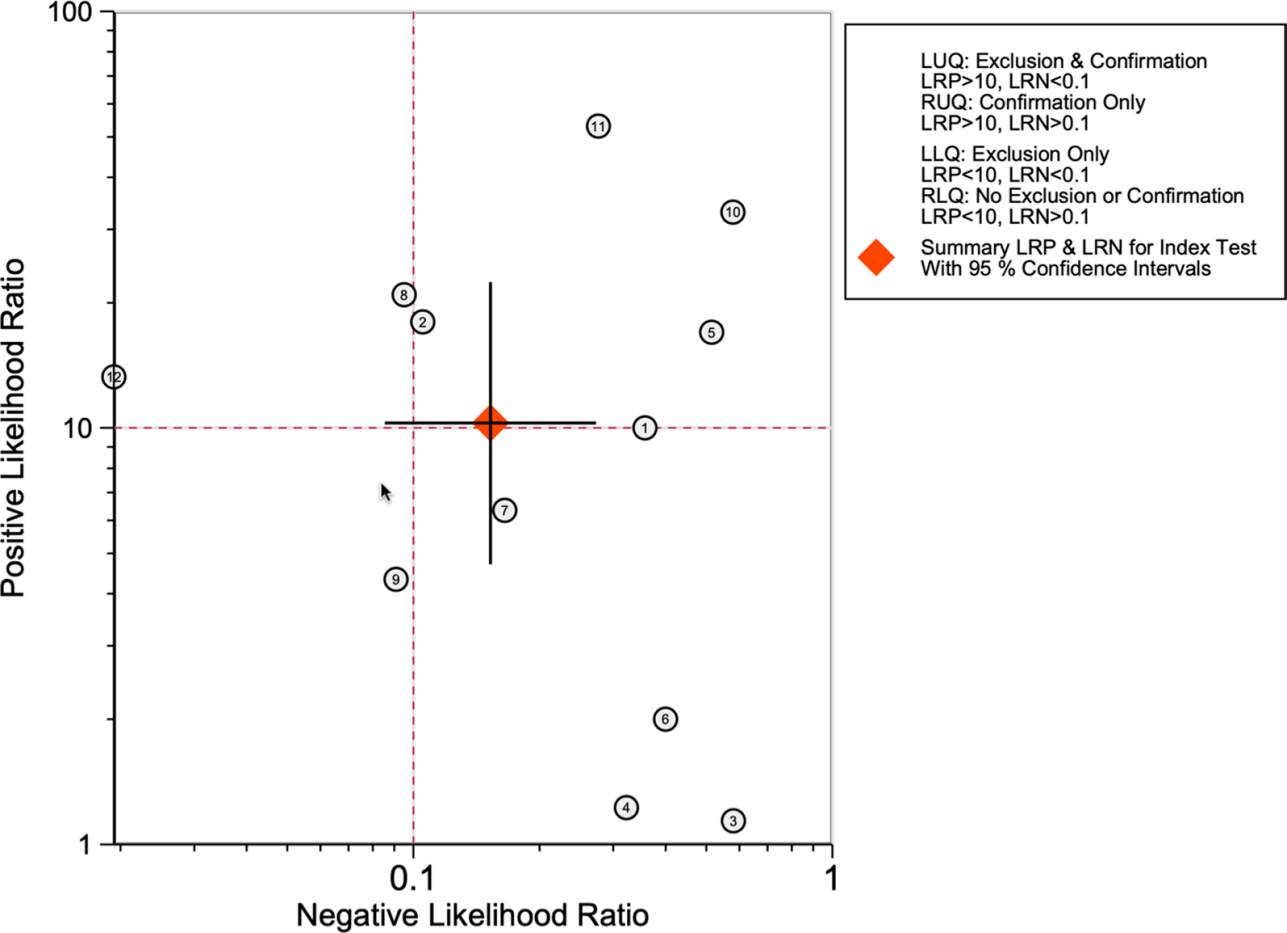
LR dot plot showing the summary of LRN and LRP and the position in the quadrants

The value of the clinical application of SPECT/CT in the diagnosis of loosening in painful knee arthroplasty was determined by analytically plotting a Fagan diagram (Fig. 7). Assuming a pretest probability of 50% for experiencing the pathological condition, SPECT/CT could accurately diagnose 92% of the patients when a positive result was present, and 12% of patients were misdiagnosed when a negative result was present. The LR dot plot shows the position in the upper right quadrant, indicating the confirmatory ability of the test in clinical practice (Fig. 8).

**Fig. 9:**
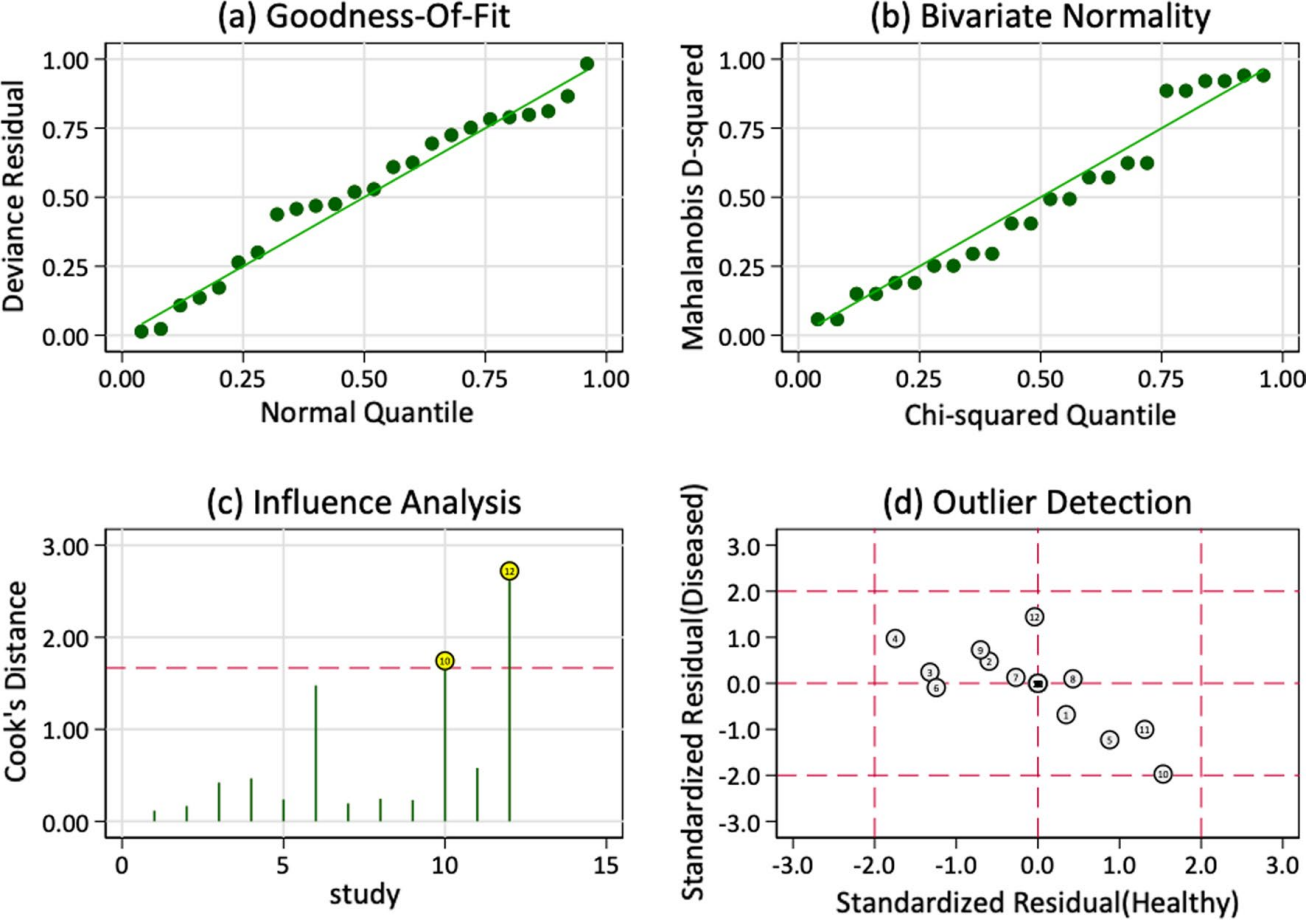
Sensitivity analysis of SPECT/CT in the diagnosis of unhappy knee arthroplasty: **a** goodness of fit; **b** Bivariate normality

The results of the goodness of fit showed that it was appropriate to select the random effects bivariate model for analysis, showing that the study had good stability (Fig. 9).

**Fig. 10:**
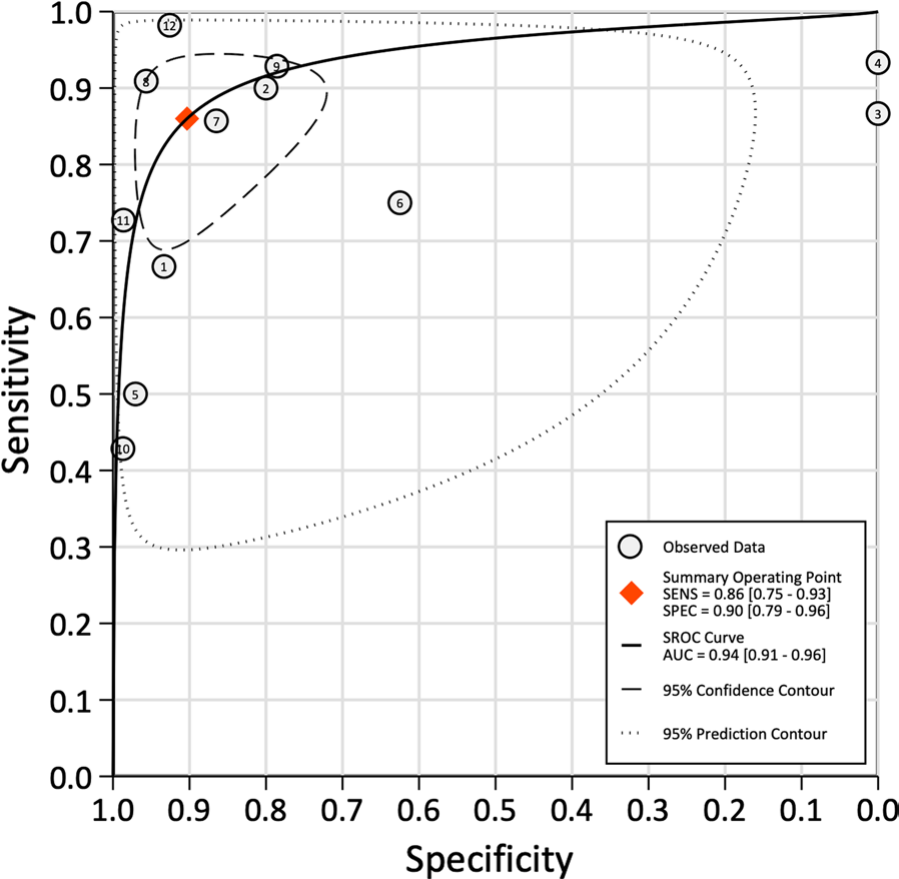
SROC showing the average sensitivity and specificity estimate of the study results with the 95% confidence region. AUC: area under the curve; SROC: summary receiver operating characteristic

The combined AUC was 0.94 (95% CI: 0.91–0.96) (Fig. 10).

**Fig. 11:**
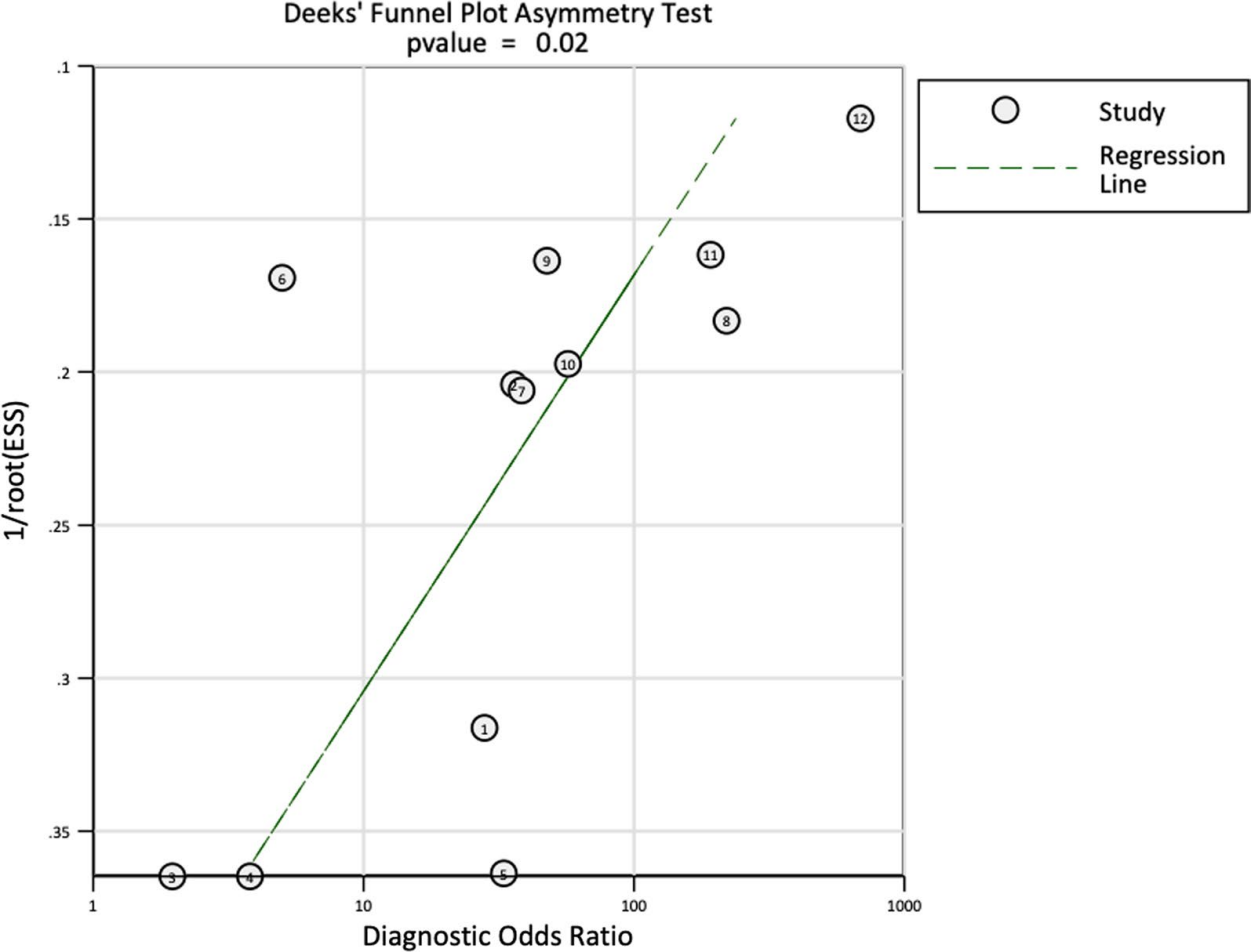
Deeks’ funnel plot asymmetry test was used to explore publication bias. ESS: effective sample size

Deeks’ symmetry was used to test for publication bias; the *P* value was not statistically significant (*P* = 0.02), indicating publication bias (Fig. 11).

The sources of heterogeneity were explored through meta-regression. The results with categorical covariates are depicted in Table 2. It was shown that the heterogeneity was related to risk of bias, the anatomical place of the prosthesis (tibial component and patella) and the route of administration (intraarticular or intravenously) (Table 2, Additional file 2: Supplement 2).

**Table 2:** Meta-regression and categorical covariates analysis for sensitivity and specificity of SPECT/CT for diagnosis of painful aseptic loosening knee prosthesis

Covariate	Parameter	Subgroup	Estimate+
Risk of bias	Sen	Some concerns	0.84 (0.56–0.96)*
		low	0.87 (0.74–0.93)*
	Esp	Some concerns	0.91 (0.69–0.98)*
		Low	0.90 (0.75–0.97)*
Intraarticular/intravenous radiotracer use	Sens	i.v	0.89 (0.79–0.95)*
		i.a	0.71 (0.40–0.90)
	Esp	i.v	0.92(0.80–0.97)*
		i.a	0.88 (0.68–0.97)*
Type	Sen	General	0.86 (0.68–0.95)*
		Femoral	0.73 (0.50–0.88)
		Tibial	0.87 (0.68–0.95)*
		Patellar	0.98 (0.85–1.00)*
	Esp	General	0.92 (0.74–0.98)*
		Femoral	0.80 (0.39–0.96)
		Tibial	0.90 (0.56–0.98)*
		Patellar	0.94 (0.41–1.00)

+ Ho: *P* = 0.5 versus Ha: *P* = 0.5/0.5

*p value < 0.05

The test accuracy of SPECT/CT according to the GRADE assessment was moderate, and after the analysis of different judgements (test accuracy, desirable and undesirable effects, certainty of the evidence of test accuracy, test effects, values, cost-effectiveness), a conditional recommendation for the use of SPECT/CT in identifying the source of knee pain after TKA was made (Additional file 3: Supplement 3).

## Discussion

This systematic review and meta-analysis showed that SPECT/CT, as a diagnostic test, has an overall sensitivity of 0.86 (95% CI: 0.75–0.93) and specificity of 0.90 (95% CI: 0.79–0.96) in diagnosing the source of pain in painful, noninfected knees after knee arthroplasty. With a + LR and -LR of 8.89 and 0.15, respectively, SPECT/CT has good accuracy in detecting the source of pain in painful knee arthroplasty patients. With a moderate certainty of evidence according to the GRADE assessment, it was possible to make a conditional recommendation for the SPECT/CT tool for localizing the source of pain in patients with painful knees after knee arthroplasty.

There are a few studies in the literature similar to this systematic review. For example, Barnsley et al. [26] compared the accuracy of different techniques (bone scan, SPECT/CT, 18-FDG-PET) in the detection of aseptic loosening and found that SPECT/CT arthrography had the best performance; however the results for SPECT/CT were obtained just from two studies comprising 61 patients: sensitivities of 0.75 (femur), 0.86 (tibia) and 1.00 (global result in one study) sensitivities of 0.63 (femur), 0.86 (tibia) and 0.93 (global result in one study) were reported, but the authors did not calculated pooled estimates. Peng et al. [27] evaluated the diagnostic performance of SPECT/CT in assessing prosthetic loosening after hip or knee replacement. Their results, although collected from gathering hip and knee replacement studies, were similar to the ones reported in this systematic review, including an AUC of 0.97, a pooled sensitivity of 0.94 (0.90–0.96) and a pooled specificity of 0.89 (0.78–0.95). With high heterogeneity in the meta-regression, they highlighted how the heterogeneity of the studies was related to factors such as the type of radiotracer used, route of administration and method of image analysis. Verberne et al. [28] reported on the intra- and interobserver agreement of nuclear imaging modalities for diagnosing infection and mechanical loosening in knee and hip arthroplasty patients. They found that SPECT/CT and FDG-PET/CT had substantial interobserver agreement for symptomatic hip arthroplasty.

The present systematic review included a broader spectrum of diagnostic studies focusing only on painful, noninfected knees after knee arthroplasty. With a medium risk of bias according to the QUADAS-2 tool, this review showed high heterogeneity between the studies, similar to previous reports [26, 27].

The LR values presented in the analysis provided support for the clinical applicability of SPECT/CT in detecting the source of pain in painful knee arthroplasty patients; SPECT/CT can clearly help clinicians make decisions regarding treatment. The SROC curve showed an AUC of 0.94 (95% CI: 0.95–0.98), confirming the good diagnostic performance of this tool.

Three authors [41, 43, 46] reported different sensitivities and specificities for each compartment, highlighting that although the values for detecting patellofemoral osteoarthritis in unresurfaced patellae were high (95% sensitivity), the performance was better in detecting loosening of the tibial and femoral components (98%, 0.90–1.00 and 0.73%, 0.39–0.94, respectively). The highest diagnostic accuracy for SPECT/CT in detecting loosening was reported by Abele et al. and Bao et al. [39, 42], who used intraarticular administration of the radiotracer ^99m^Tc sulphur colloid with a sensitivity and negative predictive value of 100%, which was superior to other imaging methods. SPECT/CT was also able to identify other sources of pain, such as patellofemoral hyper pressure, instability and malalignment of the components, with findings confirmed by intraoperative or clinical follow-up [21, 47].

The majority of authors applied a systematic method by using an image scheme protocol that allowed them a) to report typical patterns of BTU for specific pathologies with high intra- and interobserver correlation and b) to use a biomechanical approach to the clinical condition.

Hirschmann et al. [22] presented the first approach to an image scheme analysis that systematically evaluated the prosthetic component position, with high inter- and intraobserver reliability for grading and localizing tracer activity (intraclass correlation coefficient (ICC) = 0.95). Using the same image scheme analysis, Iranpour et al. [48] showed a high ICC between BTU and rotational/alignment measurements in the femoral and tibial components. Awengen et al. [49] demonstrated a significant correlation between the positions of the knee components and BTU. Konala et al. [50] reported a clinically relevant change in the diagnostic and treatment strategy after SPECT/CT in 83% of their patients. They also demonstrated that rotational and alignment abnormalities generate specific patterns of BTU around the components and that malpositioning of prosthetic components is a key factor leading to TKA failure. Slevin et al. [51] showed a significant correlation between valgus alignment of the femoral TKA and increased BTU at the lateral patellar regions (*P* < 0.05). Verschueren et al. [52] reported that when linking prosthesis rotational alignment with blood pool SPECT uptake scores, femoral internal rotation was associated with higher uptake in the posteromedial (*P* = 0.042) and anterolateral regions (*P* = 0.016) of the femur. They also found that an increased blood pool at the internal tibial plateau was related to a sixfold–12-fold increase in the need for revision surgery. Rasch et al. [47] found a significant correlation between valgus alignment of the femoral component and increased BTU in the lateral patellar areas (*P* < 0.005). The use of this image scheme was also important for defining particular BTU patterns associated with specific conditions, such as malpositioning of the components, maltracking, patellar overloading, patellar osteoarthritis and loosening, which led the clinician to change the initial diagnosis in up to 85% of the patients [40, 41, 46]. It is important to highlight a recent report by Mathis et al. [21], which showed a significant correlation (*P* < 0.05) between different patterns of BTU and the position of the TKA components together with different pain patterns, enhancing the importance of the component position as a potential source of pain in painful knee arthroplasty patients. The authors concluded that component positioning-related pathologies accounted for the greatest proportion of cases, followed by patella-related problems and instability.

The benefits of using a consistent model for systematically analysing patients with painful prostheses are as follows: the possibility of comparing and following results in clinical studies and establishing the correct diagnosis; the possibility of identifying a clinically unsuspected origin of pain, leading to better patient management; and the ability to obtain an accurate diagnosis with a significant impact on costs to health systems.

SPECT/CT offers functional data about bone turnover patterns together with biomechanical information in TKA patients [49], helping to select appropriate clinical management options and assess the need for surgical treatment [41]. In a cost-effectiveness study, Van den Wyngaert et al. [53] translated these clinical benefits into economic outcomes, reporting that SPECT/CT resulted in lower costs ($1,867,695 less) than CT alone over three years or $622 per patient/year. Cost-effectiveness appears to be primarily driven by better diagnostic accuracy, thus avoiding undue surgeries and ad hoc medical management; the use of SPECT/CT was associated with both lower costs and improved quality of life per year.

In summary, current evidence supports the use of SPECT/CT for identifying the source of pain in painful knees after knee arthroplasty through the use of BTU patterns, where a negative scan is highly predictive of the absence of major complications, particularly in painful prostheses. The method provides combined structural, mechanical and functional information, showing benefits for establishing a diagnosis and providing guidance for further treatment. A number of justifications for the use of SPECT/CT in clinical practice in painful knee arthroplasty can be defined, including the degree to which this tool can impact the patient outcome; the reasonability of its costs; its non-invasive nature; the ability to perform the scan at different time points in the course of the disease; the ability to provide additional information that can change adherence or the nature of the intervention given; the potential to speed up decision-making and thus improve patient quality of life; the ability to correct the initial clinical suspicion for tibial or femoral component loosening and provide different underlying causes of persistent knee pain after TKA (patellofemoral overloading, patellar malalignment, instability, degenerative patellofemoral changes, amongst others); and the ability to detect component loosening that was not initially clinically suspected.

There are implications in the use of SPECT/CT for clinicians and policymakers:

Improved diagnostic accuracy: This hybrid image system (SPECT/CT) can improve the accuracy of diagnosis, leading to better patient outcomes and potentially reducing the need for additional imaging studies or equivocal treatments.

Increased efficiency: SPECT/CT can modernize the diagnostic process, facilitating clinicians in making more accurate diagnoses in a shorter amount of time with potential cost savings and improved patient care.

Enhanced treatment planning: SPECT/CT can provide valuable information for surgery planning, allowing surgeons to improve patient outcomes.

For policymakers, the use of SPECT/CT in painful knee arthroplasty may have implications for resource allocation and healthcare budgeting.

Because a revision surgery is not recommended in cases of unexplained pain in TKA, an optimal diagnostic algorithm becomes essential to identify all possible causes of pain. It should be based on medical history, clinical examination, microbiological work-up and detailed imaging analysis. Standardized conventional radiographs are mandatory to identify component malposition, polyethylene wear, component over- or under-sizing, overfilling or gross loosening [54]. Only when medical history, physical examination or standard radiological investigations cannot explain the symptoms, SPECT/CT is recommended to evaluate component positioning in 3D CT and the metabolic characteristics of bone structures and components, in order to establish differential diagnosis such as: loosening/wear, malposition/malsizing, patellofemoral osteoarthritis, stiffness, extensor mechanism, instability, infection and other potential causes. SPECT/CT is able to change the initial diagnosis in a high percentage of patients, leading to more targeted, conservative and effective treatments. The diagnostic benefits of the technique are clear combining mechanical, structural and biological information and in daily practice it should be implemented as part of the routine diagnostic algorithm for patients with painful knee arthroplasty.

Some limitations need to be acknowledged. First, relevant information could have been missed by restricting the search of publications to those written in English, Italian and Spanish. Likewise, the exclusion of unpublished data, ongoing studies and existing studies for which relevant data could not be obtained might have led to publication bias.

Second, the p value of Deeks’ symmetry test for publication bias was 0.02, and although it could be explained by publication bias concerns, the nature of this statistical method forces us to interpret it with caution and to consider other explanations for the small-study effects and heterogeneity, such as the influence of covariates or chance. Third, there was high heterogeneity in this study, with one of the major sources of bias being the use of different comparators, diverse surgical techniques and other factors belonging to the non-threshold effect model such as type of risk of bias, route of administration of the radiotracer and the anatomical place of the evaluated prosthesis; although the meta-regression model showed significant differences in the results, it is important to recognize the impact of the of the low sample size of the series; the results should be interpreted with caution and need to be validated with future studies including larger patient samples. Fourth, it was not possible to identify sufficient publications regarding SPECT/CT as a diagnostic test, so the results reported in this review came from a limited number of patients. Fifth, for some of the reviewed series, the sample size was small, and the authors did not explicitly calculate its size or establish a conceptual hypothesis.

As mentioned above, few systematic reviews related to this topic have been published, and although the results reported in this current review are similar to previous reports [27], it is possible to identify some differences and strengths that can add significant value to the previously published data. First, the results came exclusively from a population with aseptic painful knee replacement with a larger sample size of patients who could be gathered for the analysis. Second, the test accuracy of SPECT/CT as diagnostic test was analysed using the GRADE assessment, yielding a moderate score and generating a conditional recommendation for the use of this imaging tool. Third, although the literature search was done on May 2022 for the purpose of this review, the database was checked once more as an upgrade on December 2022; however, no new articles were identified.

### Future perspectives

To improve the strength of the current evidence, it is necessary to conduct studies with a larger number of patients with a multicentre and prospective design to be able to compare different populations and types of arthroplasty models and improve confidence in the accuracy of SPECT/CT. Improvements in device technology, such as the introduction of metallic beam artefact suppression techniques and the possibility of using new software for quantification purposes in nuclear medicine, could lead to more accurate results and could provide new standards for image evaluation; it will be necessary to conduct studies with the new standards of the novel technology.

## Conclusions

The evidence summarized in this systematic review and meta-analysis highlights the performance of SPECT/CT in diagnosing the source of pain in painful, noninfected knees after knee arthroplasty. With high heterogeneity between published studies, the best evidence available to date shows that SPECT/CT has high sensitivity and specificity in identifying the source of pain in noninfected knees after knee arthroplasty, particularly in cases of loosening, patellofemoral disorders and component malalignment. Including this imaging tool in the diagnostic flow of painful knee arthroplasty will have significant clinical repercussions: changing the initial diagnosis, identifying or excluding different causes of painful knee arthroplasties, guiding subsequent treatment and positively impacting the final clinical outcome.

## Supplementary Information

**Additional file 1: Supplement 1** Search strategy and search strategy database.

**Additional file 2: Supplement 2** Forest plot meta-regression.

**Additional file 3: Supplement 3** GRADE score analysis.

**Additional file 4: Supplement 4** PRISMA checklist.

**Additional file 5: Supplement 5** TP, TN, FP, FN data from papers.

## Data Availability

Not applicable.

## Abbreviations

SPECT: Single-photon emission computed tomography
XR: Conventional X-rays
CT: Computed tomography
MRI: Magnetic resonance imaging
BTU: Bone tracer uptake
SEN: Sensitivity
SPE: Specificity
LRP: Positive likelihood ratio
LRN: Negative likelihood ratio
DOR: Diagnostic odds ratio
CI: Confidence interval
SROC: Summary receiver operating characteristics
LR: Likelihood ratio
AUC: Area under the curve
ESS: Effective sample size
