# Supplementary figures and images for: The role of SPECT/CT in painful, noninfected knees after knee arthroplasty: a systematic review and meta-analysis—a diagnostic test accuracy review
Source: J Orthop Surg Res. 2023 Mar 21;18:223. doi: 10.1186/s13018-023-03687-8 (PMC10031962; doi:10.1186/s13018-023-03687-8)

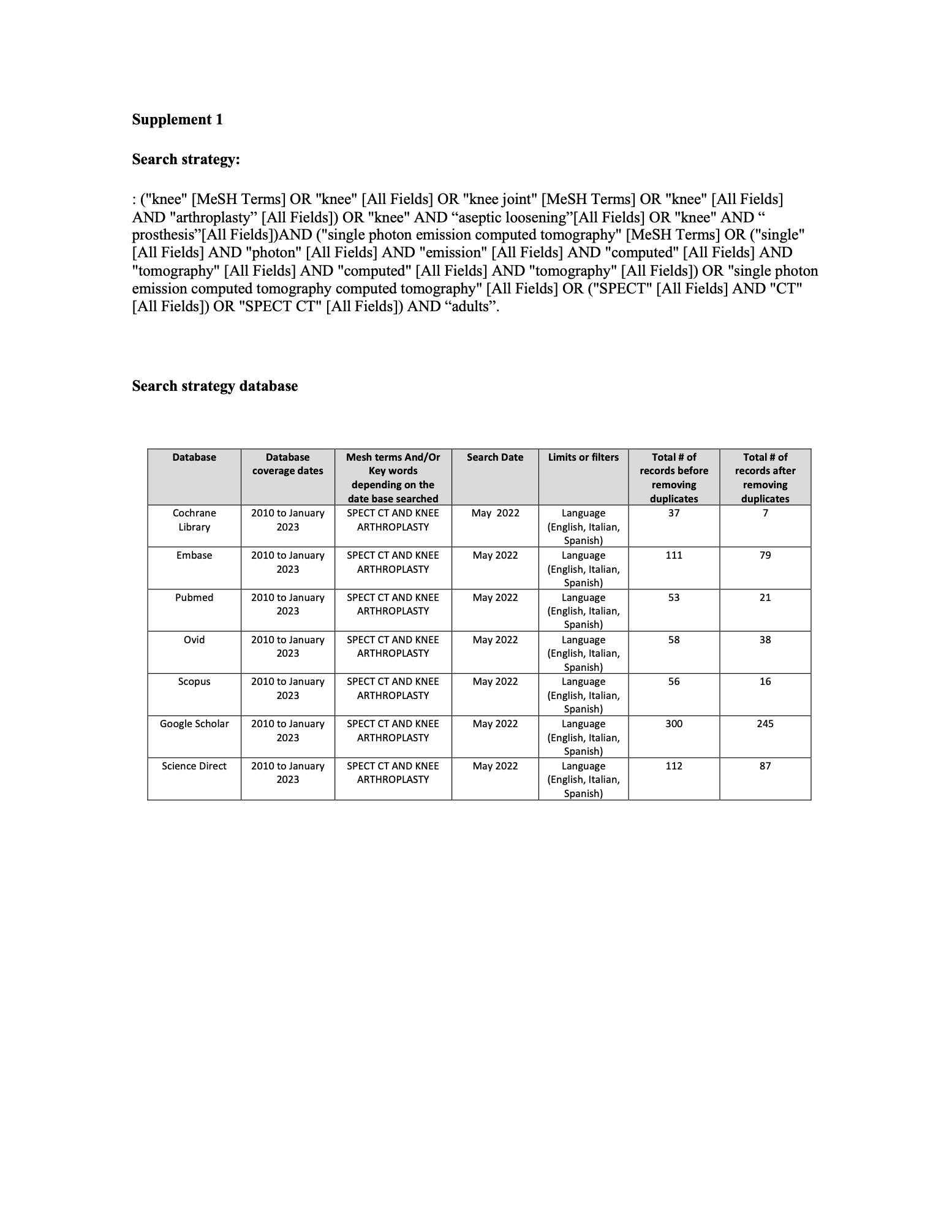

Supplement: Supplementary file 1 — Additional file 1: Supplement 1 Search strategy and search strategy database. [file 13018_2023_3687_MOESM1_ESM.tiff]

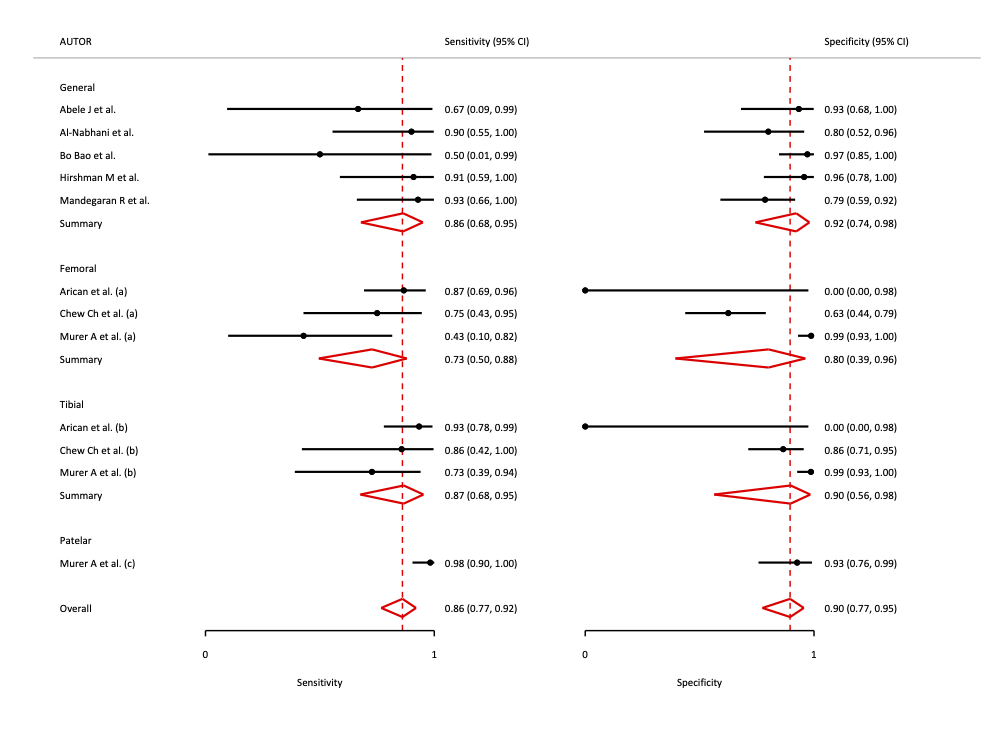

Supplement: Supplementary file 2 — Additional file 2: Supplement 2 Forest plot meta-regression. [file 13018_2023_3687_MOESM2_ESM.tiff]

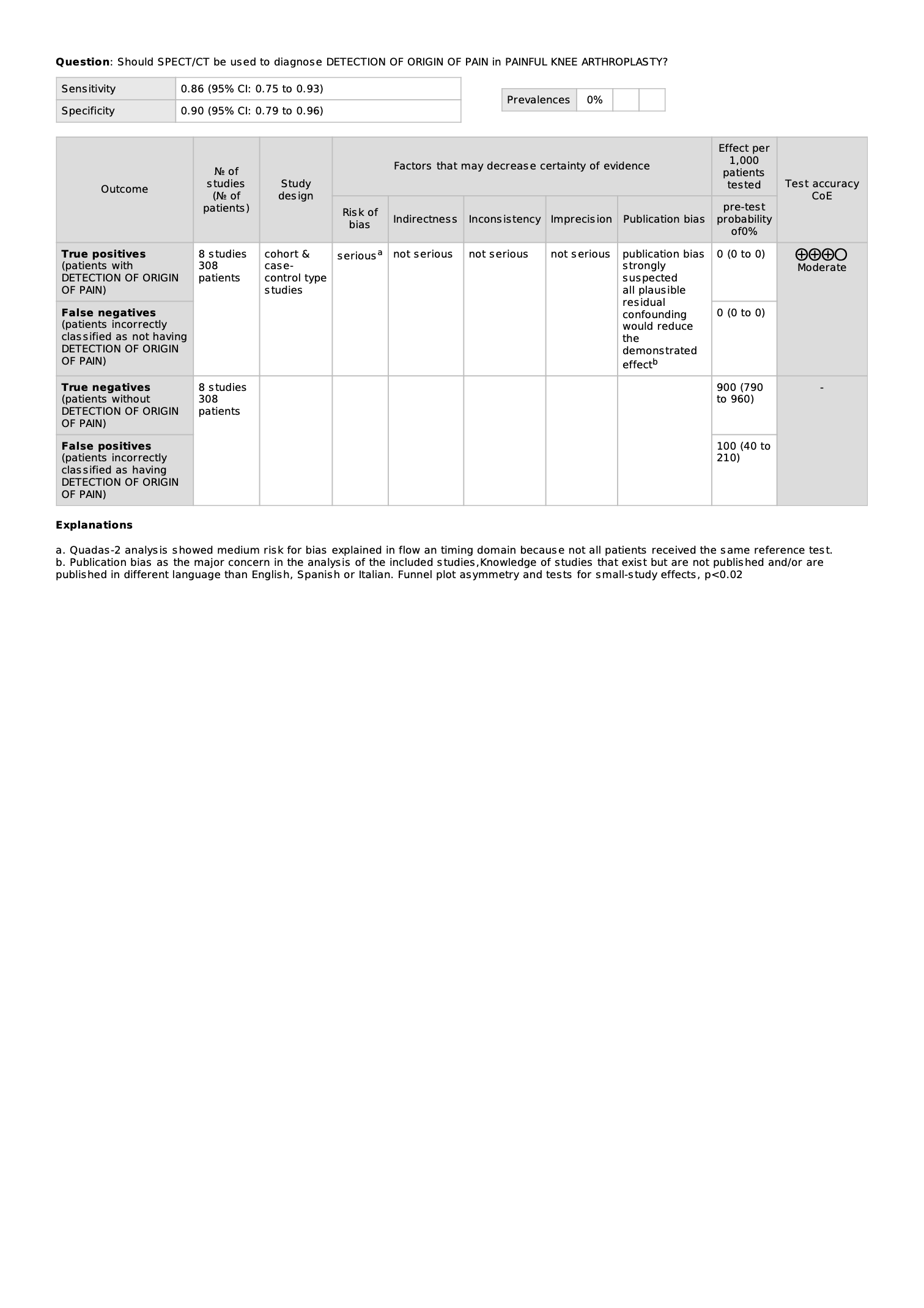

Supplement: Supplementary file 3 — Additional file 3: Supplement 3 GRADE score analysis. [file 13018_2023_3687_MOESM3_ESM.tiff]
