# Supplementary material for: The role of SPECT/CT in painful, noninfected knees after knee arthroplasty: a systematic review and meta-analysis—a diagnostic test accuracy review
Source: J Orthop Surg Res. 2023 Mar 21;18:223. doi: 10.1186/s13018-023-03687-8 (PMC10031962; doi:10.1186/s13018-023-03687-8)
Supplement: Supplementary file 4 — Additional file 4: Supplement 4 PRISMA checklist. [file 13018_2023_3687_MOESM4_ESM.pdf]

PRISMA-DTA Checklist

Section/topic	#	PRISMA-DTA Checklist Item	Reported on page #
**TITLE / ABSTRACT**			
Title	1	**The role of SPECT/CT in painful non-infected knees after knee arthroplasty : systematic review and meta analysis, a diagnostic test accuracy review.**	1
Abstract	2	**Purpose:** For improving for the clinicians the available information on the current written evidence related to the diagnostic approach in aseptic painful knee arthroplasty, it was conducted this systematic review with the primary aim to analyse the best evidence regarding to diagnostic tests, about ^{99m}Tc-phosphate SPECT/CT in the evaluation of unhappy non infected knee arthroplasty.<br>**Methods**: Embase, PubMed, Google Scholar, Ovid, Scopus, Science Direct, and the Cochrane Database of Systematic Reviews were searched from database inception to May 2022 following PRISMA guidelines. As a primary outcome, it was defined the role of SPECT/CT in the diagnostic approach to noninfected painful knee arthroplasty; as a secondary objective, there were described non-infection-related factors linked to painful knee arthroplasty. Pooled sensitivity, specificity, positive likelihood ratio, negative likelihood ratio, diagnostic odds ratio and other indicators were calculated; ROC analysis and the summary of AUCs from the included studies were reported. A Fagan plot, likelihood ratio plot and Deeks funnel plot were generated and analysed. Methodological quality was assessed using the QUADAS-2. Certainty of evidence assessment was made by using GRADE guidance.<br>**Results**: A total of 493 publications were identified, of which 8 met the inclusion criteria. The pooled sensitivity and specificity of SPECT/CT for diagnosing the source of pain in painful knee prostheses were 0.86 (95% CI: (0.75-0.93) and 0.90 (95% CI: 0.79-0.96), respectively, with pooled +LR and -LR values of 8.9 (95% CI: 4.11-19.19) and 0.15 (95% CI: 0.09-0.28), respectively. The pooled diagnostic odds ratio was 57.35, and the area under the curve was 0.94. Based on different bone tracer uptake patterns, SPECT/CT identified different sources of pain, such as loosening of the prosthetic components, patellofemoral overloading, instability, malalignment of the components, and degeneration of the patellofemoral compartment. Risk of bias under QUADAS-2 was medium. Certainty of evidence was moderate under GRADE assessment.<br>**Conclusion**: As a diagnostic tool, SPECT/CT has been proven to have high sensitivity and specificity in identifying the source of pain in unhappy knees after knee arthroplasty, particularly in cases of loosening, patellofemoral disorders and component malalignment. It has significant clinical repercussions: changing the initial diagnosis, identifying or excluding different causes of painful knee arthroplasties, guiding subsequent treatment, and positively impacting the final clinical outcome. (level of evidence III). Moderate recommendation according to GRADE assessment.	1
**INTRODUCTION**			
Rationale	3	Because the amount of clinical and imaging information included in the evaluation of unhappy knee prosthesis patients is crucial for a better diagnostic approach, it is important to know the performance of different imaging tools to determine their role as part of a diagnostic work-up in this clinical scenario. Published evidence on SPECT/CT application in the diagnostic approach of the painful knee after knee arthroplasty is scarce, has increased in recent years, and the utility of this modality has been demonstrated in different scenarios where conventional imaging tools have limitations; it was considered important to analyse the current evidence about diagnostic tests regarding to ^{99m}Tc-phosphate SPECT/CT in the evaluation of unhappy knee arthroplasty, because, although the literature has shown the	2

		potential of this technique improving the accuracy for detecting the source of pain, its place as part of a diagnostic work-up has not been clearly supported.	
Clinical role of index test	D1	Technological improvements of CT and nuclear medicine devices have led to higher-resolution images, permitting better qualitative and quantitative analysis of bone tracer uptake (BTU) with superior anatomical correlation. Hybrid SPECT/CT, with a radiation burden of only 2-4 mSv, has emerged as a hybrid imaging tool with important advantages for the diagnostic approach to the painful knee after arthroplasty. Some of these advantages are as follows: a) offering the anatomical detail and spatial resolution of CT, enhancing the specificity of SPECT ; b) detecting osteoblastic activity between multiple time points, providing insights into treatment outcomes for individual patients; c) giving guidance for choosing among treatment options in patients with postoperative knee pain ; d) revealing stress in the subchondral bone, which correlates with the source of pain and e) detecting functional changes before abnormalities can be seen by RX, CT or MRI	2
Objectives	4	This systematic review aims to analyse the current evidence about diagnostic tests accuracy of ^{99m}Tc-phosphate SPECT/CT in the evaluation of unhappy knee arthroplasty. A meta-analysis on quantitative methods was also conducted where possible. As a primary outcome, it was defined the role of SPECT/CT in the diagnostic approach to noninfected painful knee arthroplasty; as a secondary objective, there wer described non-infection-related factors linked to painful knee arthroplasty.	2
**METHODS**			
Protocol and registration	5	This protocol was approved by the ethical committee of our institution with registration number CEIFUS 1096-21.<br>This review was registered in Prospero under code CRD42022320457.<br>.	1
Eligibility criteria	6	**Criteria for considering studies for this review**<br>**Types of studies**: This review included full-text reports of systematic reviews, observational studies and prospective and retrospective cohort studies, the data of which had the potential to be used for analysis purposes in the category of diagnostic studies. Studies with a sample size larger than 10 cases which allowed the possibility of constructing a 2x2 table<br>**Types of participants**: adult patients with painful knees after primary (partial or total) or revision knee arthroplasty who had undergone to bone SPECT/CT scintigraphy as part of their diagnostic work-up.<br>**Types of intervention**: ^{99}mTc HMDP / ^{99}mTc HDP/ ^{99}mTc MDP intravenously or intra articular SPECT/CT<br>**Types of study outcome**: The final diagnostic was related to aseptic painful knee in arthroplasties. The gold standard in the study was either the result of an intraoperative finding, histological examination, clinical outcome or a comparable standard result<br>**Studies were excluded** if the focus was on oncology or infection-related applications or in which the emphasis was placed on aspects related to generators, radiochemistry, animal models, experimental reports or physics. Descriptive studies focused solely on the pattern of bone tracer uptake (BTU) in which the authors did not use any comparator to infer the explanation of the findings were also excluded. Small-sample-size studies with fewer than 10 patients were not included, as the minimum requirements for analysis in this review were not met. -Reviews, case reports, conference presentations and so on. -Inability to separate results of knee from other prosthesis. -Inability to separate results from different pathologies (e.g septic from aseptic loosening).<br>**Years considered**: 2010-2022.	3

PRISMA-DTA Checklist

<table>
<tr><td></td><td></td><td>Language considered: English, Spanish, Italian.</td><td></td></tr>
<tr><td>Information sources</td><td>7</td><td>The Cochrane Central Register of Controlled Trials (CENTRAL) published in the Cochrane Library, Embase, PubMed, Google Scholar, Ovid, Scopus and Science Direct were all searched. The language was restricted to English, Italian and Spanish. We searched grey literature through clinicaltrials.gov and a summary of conferences. When possible, authors were contacted for clarification. All relevant studies published from 2010 to May 2022 were included for the analysis purpose.</td><td></td></tr>
<tr><td>Search</td><td>8</td><td>Search strategy:
: ("knee" [MeSH Terms] OR "knee" [All Fields] OR "knee joint" [MeSH Terms] OR "knee" [All Fields] AND "arthroplasty" [All Fields]) OR "knee" AND “aseptic loosening”[All Fields] OR "knee" AND “ prosthesis”[All Fields])AND ("single photon emission computed tomography" [MeSH Terms] OR ("single" [All Fields] AND "photon" [All Fields] AND "emission" [All Fields] AND "computed" [All Fields] AND "tomography" [All Fields] AND "computed" [All Fields] AND "tomography" [All Fields]) OR "single photon emission computed tomography computed tomography" [All Fields] OR ("SPECT" [All Fields] AND "CT" [All Fields]) OR "SPECT CT" [All Fields]) AND “adults”.
<table>
<tr><th>Database</th><th>Database coverage dates</th><th>Mesh terms And/Or Key words depending on the date base searched</th><th>Search Date</th><th>Limits or filters</th><th>Total # of records before removing duplicates</th><th>Total # of records after removing duplicates</th></tr>
<tr><td>Cochrane Library</td><td>2010 to January 2023</td><td>SPECT CT AND KNEE ARTHROPLASTY</td><td>May 2022</td><td>Language (English, Italian, Spanish)</td><td>37</td><td>7</td></tr>
<tr><td>Embase</td><td>2010 to January 2023</td><td>SPECT CT AND KNEE ARTHROPLASTY</td><td>May 2022</td><td>Language (English, Italian, Spanish)</td><td>111</td><td>79</td></tr>
<tr><td>Pubmed</td><td>2010 to January 2023</td><td>SPECT CT AND KNEE ARTHROPLASTY</td><td>May 2022</td><td>Language (English, Italian, Spanish)</td><td>53</td><td>21</td></tr>
<tr><td>Ovid</td><td>2010 to January 2023</td><td>SPECT CT AND KNEE ARTHROPLASTY</td><td>May 2022</td><td>Language (English, Italian, Spanish)</td><td>58</td><td>38</td></tr>
<tr><td>Scopus</td><td>2010 to January 2023</td><td>SPECT CT AND KNEE ARTHROPLASTY</td><td>May 2022</td><td>Language (English, Italian, Spanish)</td><td>56</td><td>16</td></tr>
<tr><td>Google Scholar</td><td>2010 to January 2023</td><td>SPECT CT AND KNEE ARTHROPLASTY</td><td>May 2022</td><td>Language (English, Italian, Spanish)</td><td>300</td><td>245</td></tr>
<tr><td>Science Direct</td><td>2010 to January 2023</td><td>SPECT CT AND KNEE ARTHROPLASTY</td><td>May 2022</td><td>Language (English, Italian, Spanish)</td><td>112</td><td>87</td></tr>
</table></td><td>3,Supplement1</td></tr>
<tr><td>Study selection</td><td>9</td><td>Study selection, data extraction and management
The extraction of study characteristics focused on citation, first author, year of publication, country of publication, study design and method, setting/context, population characteristics, exposure, radiotracer used and route of administration, image interpretation, reference standard, sample size, operative characteristics of the tests in terms of sensitivity (SEN) and specificity (SPE).
Data were extracted separately by the authors using a standardized extraction tool; the decision to include or exclude</td><td>3,4</td></tr>
</table>

		an article was made by consensus reading among LKA, NH (experts in the topic). The aforementioned search strategy was used to obtain the titles and abstracts of studies with potential relevance to the review. LKA, and NH independently screened the titles, abstracts, and full texts for eligibility. It was ensured that multiple studies reporting on the same patient population were excluded. Therefore, the duplicate records were rechecked in another peer review round. Reviewers resolved disagreements through discussion or, if needed, by adjudication of a third reviewer (LFR). Differences were resolved by consensus.	
Data collection process	10	The method of data extraction from reports was independently. Data from authors when necessary were obtained by email.	3
Definitions for data extraction	11	The extraction of study characteristics focused on citation, study design and method, setting/context, population characteristics, exposure, image interpretation, reference standard, sample size, operative characteristics of the tests in terms of sensitivity (SEN) and specificity (SPE).	3
Risk of bias and applicability	12	The methodological quality and possible bias of the included studies were assessed using R 4.0.1 based on QUADAS-2. The quality of the included literature was evaluated for risk of bias (4 entries for patient selection, index test, reference standard, and flow and timing) and clinical applicability (3 entries for patient selection, index test, and reference standard)	4
Diagnostic accuracy measures	13	Statistical analysis was performed using Stata 17MP software (StataCorp, College Station, TX) to calculate sensitivity (SEN), specificity (SPE), the positive likelihood ratio (LRP), the negative likelihood ratio (LRN), and the diagnostic odds ratio (DOR) and its 95% confidence interval (CI). Finally, the forest map, summary receiver operating characteristic (SROC), Fagan's line diagram, and likelihood ratio (LR) dot plot were generated, and the area under the SROC curve (AUC) was calculated.	4
Synthesis of results	14	A total of 8 publications as diagnostic tests were analysed for inclusion and quantitative synthesis; a total population of 308 patients, which were analysed using the QUADAS-2 tool.<br>The overall risk for the studies evaluated using the QUADAS-2 tool was medium. The most frequent bias was verification bias, as not all patients received the same reference test; the final diagnosis regarding the cause of pain was based on intraoperative findings and clinical follow-up assessing the response to treatment. Four authors reported that SPECT/CT changed the clinical diagnosis and final treatment from 65% to 85% of all cases. The best diagnostic performance with the highest sensitivity and negative predictive values was found for loosening of the prosthetic components when the bone radiotracer ^{99}mTc sulfur colloid was used intraarticularly (SPECT/CT arthrography).<br>The test accuracy of SPECT/CT under GRADE assessment was moderate which after the analysis of different judgements (test accuracy, desirable and undesirable effects, certainty of the evidence of test accuracy, test's effects, values, cost-effectiveness, etc) the type of recommendation was conditional recommendation for the use of SPECT/CT.	7

PRISMA-DTA Checklist

<table>
<tr><th>Section/topic</th><th>#</th><th>PRISMA-DTA Checklist Item</th><th>Reporte on pag #</th></tr>
<tr><td>Meta-analysis</td><td>D2</td><td>The I^2 value of SEN was 62.71, and that of SPE was 84.98 The combined SEN of SPECT/CT for loosening was 0.86 (95% CI: 0.75-0.93), the combined SPE was 0.90 (95% CI: 0.79-0.96), the combined PLR was 8.9 (95% CI: 4.71-19.19), the combined NLR was 0.15 (95% CI: 0.09-0.28), and the combined DOR was 57.35 (95% CI: 25.42-146.73. The LR dot plot shows the position in the upper right quadrant, indicating the confirmatory ability of the test in clinical practice. The combined AUC was 0.94 (95% CI: 0.91-0.96). Deeks' symmetry was used to test for publication bias; the P value was not statistically significant (p=0.02), indicating publication bias .
The presence of a threshold effect was examined by calculating the Spearman correlation coefficient between the log of sensitivity and the log of(1-specificity). Heterogeneity due to nonthreshold effects was assessed by the Q-test (p<0.10indicates heterogeneity among studies) and I^2 test (if I^2 >50%, the heterogeneity among studies is large); a random effect model was used to combine the effect sizes. Deeks' funnel plot was used to determine whether there was publication bias in the included studies; when p>0.05, publication bias was considered absent. The stability of the results was tested by sensitivity analysis.</td><td>6,7</td></tr>
<tr><td>Additional analyses</td><td>16</td><td>The sources of heterogeneity were explored through meta regression. The results with categorical covariates show that the heterogeneity was related to risk of bias, the anatomical place of the prosthesis (tibial component and patella) and the route of administration (intra articular or intravenously.</td><td>7</td></tr>
<tr><td colspan="3">RESULTS</td><td></td></tr>
<tr><td>Study selection</td><td>17</td><td>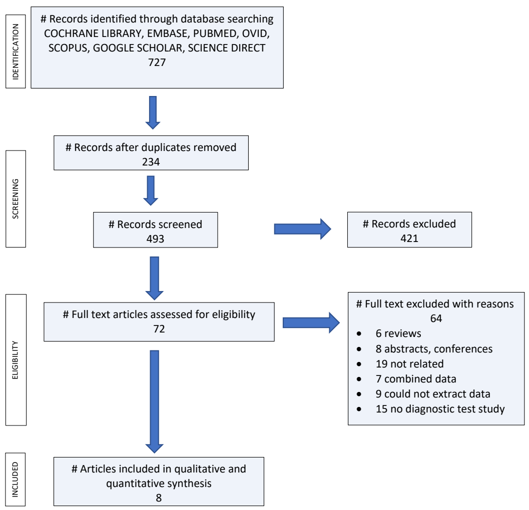

PRISMA FLOW CHART</td><td>Fig1</td></tr>
</table>

PRISMA-DTA Checklist

Section	#	Content	Reported on page
Study characteristics	18	See study characteristics table below	
Risk of bias and applicability	19	See figures below	Fig,2,3
Results of individual studies	20	See figures below	Fig,4,1

Study characteristics (18)

Author	Participant characteristics	Clinical setting	Study design	Target condition definition	Index test	Reference standard	sample size	funding sources
Abele	adults	unhappy knee arthroplasty	Retrospective	source of pain in painful knee arthroplasty	SPECT/CT	intraoperative Findings and clinical follow up	17	No
AL-Nabhani	adults	unhappy knee arthroplasty	Retrospective	source of pain in painful knee arthroplasty	SPECT/CT	intraoperative Findings and clinical follow up	24	Department of Health's National Institute for Health Research (NIHR) Biomedical Research Centres' funding scheme.
Arican	adults	unhappy knee arthroplasty	Retrospective	source of pain in painful knee arthroplasty	SPECT/CT	Intraoperative Findings	30	No
Bo Bao	adults	unhappy knee arthroplasty	Retrospective	source of pain in painful knee arthroplasty	SPECT/CT	intraoperative Findings and clinical follow up	36	No
Chew Ch	adults	unhappy knee arthroplasty	Retrospective	source of pain in painful knee arthroplasty	SPECT/CT	Intraoperative Findings	44	No
Hirschmann	adults	unhappy knee arthroplasty	Prospective	source of pain in painful knee arthroplasty	SPECT/CT	Intraoperative Findings	33	Research grant from Deutsche Arthrose-Hilfe e.V., Frankfurt, Germany.
Mandegaran	adults	unhappy knee arthroplasty	Retrospective	source of pain in painful knee arthroplasty	SPECT/CT	intraoperative Findings and clinical follow up	41	No
Murer	adults	unhappy knee arthroplasty	Retrospective	source of pain in painful knee arthroplasty	SPECT/CT	Intraoperative Findings	83	No

Risk of bias and applicability (19)

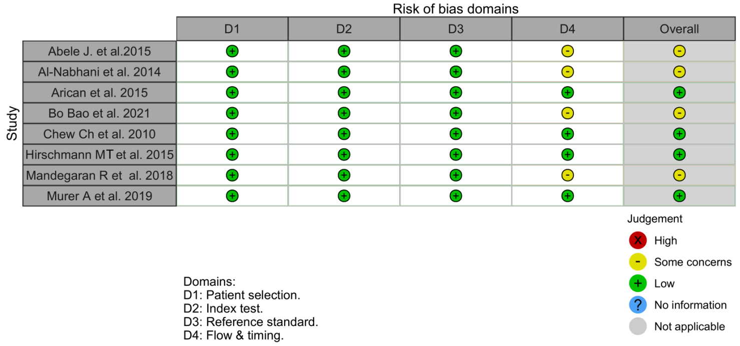

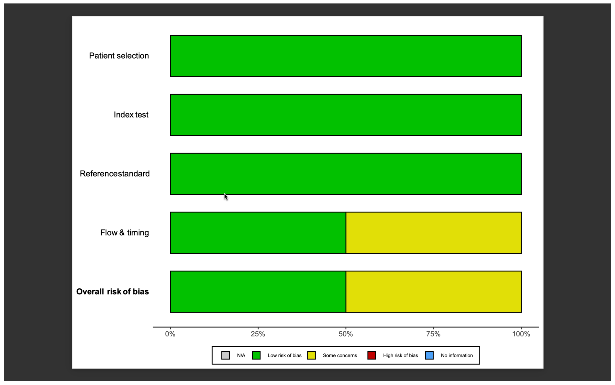

Results of individual studies (20)

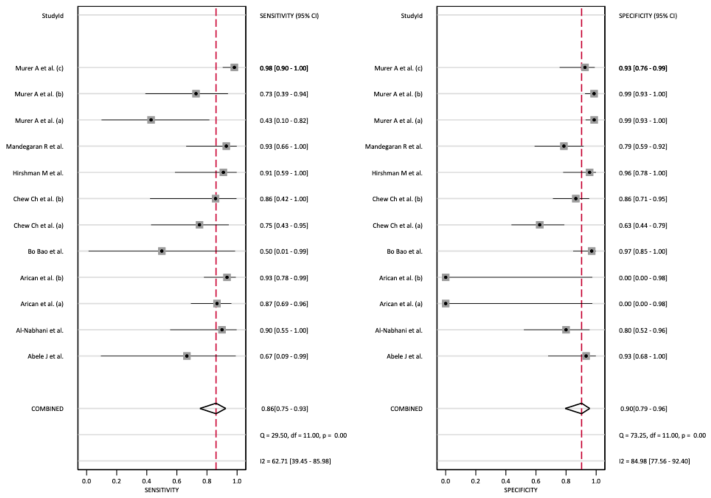

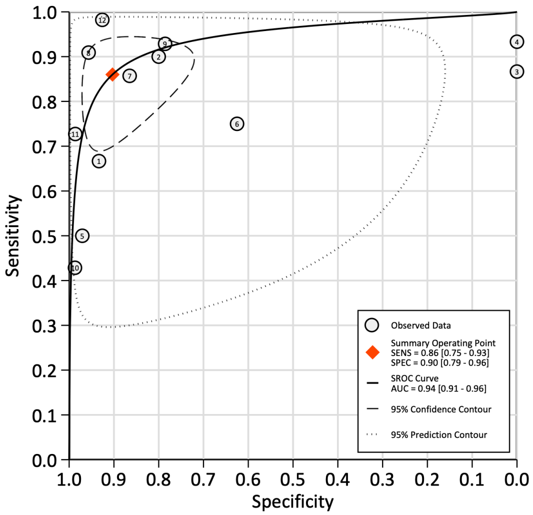

PRISMA-DTA Checklist

Synthesis of results	21	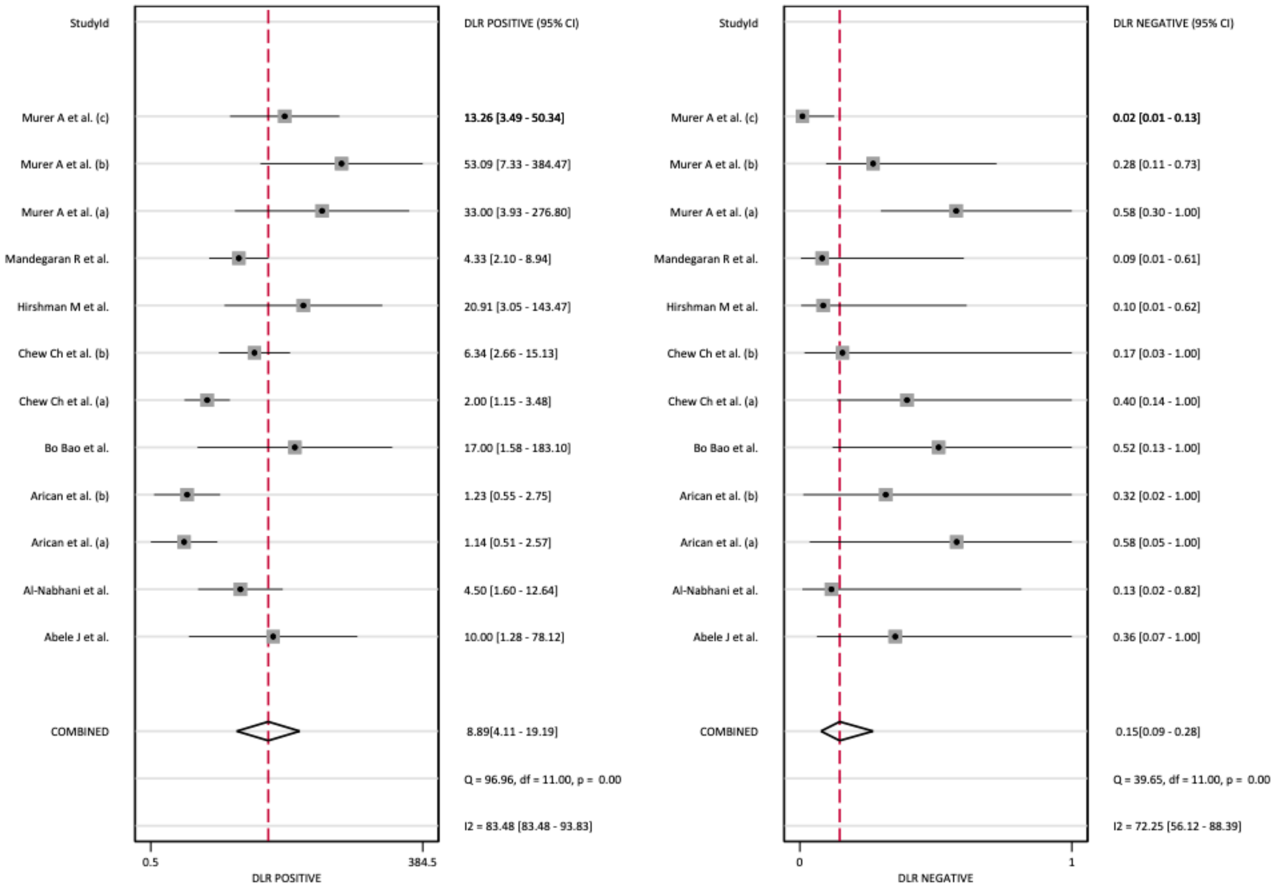<br>.	Fig6

PRISMA-DTA Checklist

Additional analysis

23

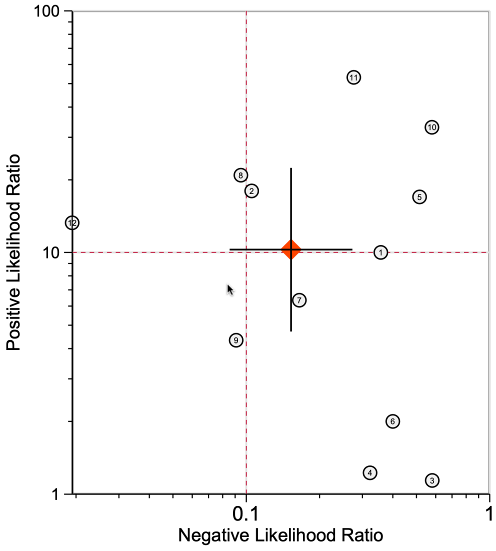

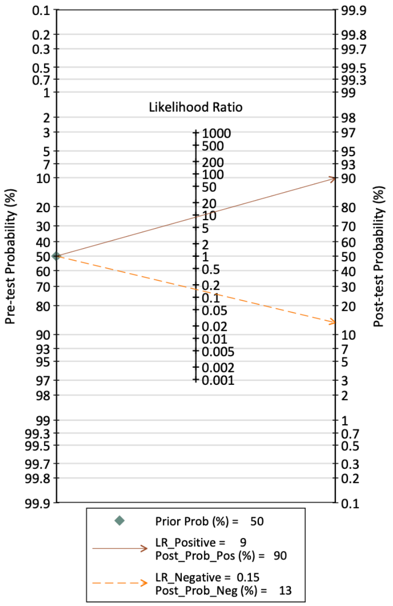

The value of clinical application of SPECT/CT for the diagnosis of source of pain of painful aseptic knee arthroplasty was determined by analytically plotting a Fagan diagram. The LR dot plot shows the position in the upper right quadrant, indicating the confirmatory ability of the test in the clinical

practice.

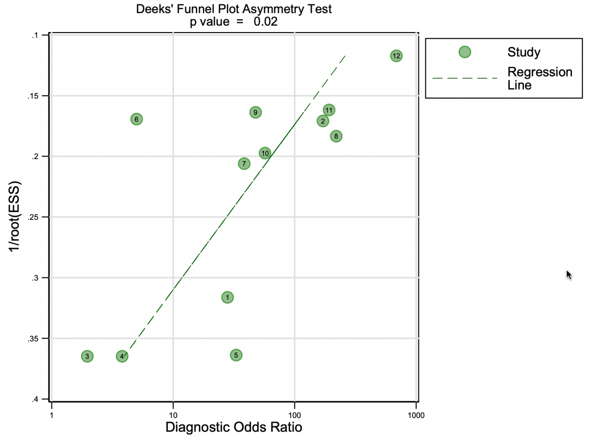

Deek's symmetry was used for publication analysis. The p value was not statistically significant (p=0.02) indicating publication bias.

Fig 7,8

11,

Suppl

2.

Covariate	Parameter	Subgroup	Estimate +
Risk of bias	Sens	Some concerns	0,84 (0,56 - 0,96) *
		Low	0,87 (0,74 - 0,93) *
	Esp	Some concerns	0,91 (0,69 - 0,98) *
		Low	0,90 (0,75 - 0,97) *
Intraarticular/intravenous radiotracer use	Sens	i.v	0,89 (0,79 - 0,95) *
		i.a	0,71 (0,40 - 0,90)
	Esp	i.v	0,92 (0,80 - 0,97) *
		i.a	0,88 (0,68 - 0,97) *
Type	Sens	General	0,86 (0,68 - 0,95) *
		Femoral	0,73 (0,50 - 0,88)
		Tibial	0,87 (0,68 - 0,95) *
		Patelar	0,98 (0,85 - 1,0) *
	Esp	General	0,92 (0,74 - 0,98) *
		Femoral	0,8 (0,39 - 0,96)
		Tibial	0,90 (0,56 - 0,98) *
		Patelar	0,94 (0,41 - 1,0)

+ NOTE: H_0: P = 0.5 vs. H_a: P ≠ 0.5

*** p value <0,05**

Meta regression and categorical covariates analysis for sensitivity and specificity of SPECT/CT for diagnosis of aseptic loosening.

**DISCUSSION**			
Summary of evidence	24	This systematic review and meta-analysis showed that SPECT/CT has an overall sensitivity of 0.86 (95% CI: 0.75-0.93) and specificity of 0.90 (95% CI: 0.79-0.96) for diagnosing the source of pain in painful, noninfected knees after knee arthroplasty. With a +LR and -LR of 8.89and 0.15, respectively, SPECT/CT has good accuracy for detecting the source of pain in unhappy knee arthroplasty patients.<br>With a medium risk of bias according to QUADAS-2, high heterogeneity between the studies, similar to previous reports, the LR values presented in this analysis, provide support for the clinical applicability of SPECT/CT in detecting the source of pain in unhappy knee arthroplasty patients.<br>The SROC curve shows an AUC of 0.94 (95% CI: 0.91-0.96), confirming the good diagnostic performance of this tool. Level of evidence III.<br>Test accuracy of SPECT/CT under GRADE assessment was moderate which after the analysis of different judgements (test accuracy, desirable and undesirable effects, certainty of the evidence of test accuracy, test's effects, values, cost-effectiveness, etc) the type of recommendation was conditional recommendation for the use of SPECT/CT.	1
Limitations	25	First, relevant information could have been missed by restricting the search of publications to those published in English, Italian and Spanish. Likewise, the exclusion of unpublished data, ongoing studies and existing studies for which relevant data could not be obtained might have led to publication bias.<br>Second, the p value of Deeks' symmetry test for publication bias was 0.02, and although it could be explained by publication bias concern, the nature of this statistical method forces us to interpret it with caution and to consider other explanations for the small-study effects and heterogeneity, such as the influence of covariates or chance. Third, there was high heterogeneity in this study, with one of the major sources of bias being the use of different comparators, diverse surgical techniques, and other factors belonging to the nonthreshold effect model such as type of risk of bias, route of administration of the radiotracer and the anatomical place of the evaluated prosthesis; although	9

		the meta regression model showed significant differences in the results, it is important to recognize the impact of the of the low sample size of the series; the results should be interpreted with caution and need to be validated with future studies including larger patient samples. Fourth, it was not possible to identify enough publications regarding SPECT/CT as a diagnostic test, so the results reported in this review came from a limited number of patients. Fifth, for some of the reviewed series, the sample size was small, and the authors did not explicitly calculate its size or establish a conceptual hypothesis.	
Conclusions	26	The evidence summarized in this systematic review and meta-analysis highlights the performance of SPECT/CT in diagnosing the source of pain in painful, noninfected knees after knee arthroplasty. With high heterogeneity between published studies, the best evidence available to date shows that SPECT/CT has high sensitivity and specificity in identifying the source of pain in noninfected knees after knee arthroplasty, particularly in cases of loosening, patellofemoral disorders and component malalignment. Including this imaging tool in the diagnostic flow of painful knee arthroplasty will have significant clinical repercussions: changing the initial diagnosis, identifying or excluding different causes of painful knee arthroplasties, guiding subsequent treatment, and positively impacting the final clinical outcome.	10
**FUNDING**			
Funding	27	Non applicable	

Adapted From: McInnes MDF, Moher D, Thombs BD, McGrath TA, Bossuyt PM, The PRISMA-DTA Group (2018). Preferred Reporting Items for a Systematic Review and Meta-analysis of Diagnostic Test Accuracy Studies: The PRISMA-DTA Statement. JAMA. 2018 Jan 23;319(4):388-396. doi: 10.1001/jama.2017.19163.

For more information, visit: **www.prisma-statement.org**.
