# Supplementary material for: The role of SPECT/CT in painful, noninfected knees after knee arthroplasty: a systematic review and meta-analysis—a diagnostic test accuracy review
Source: J Orthop Surg Res. 2023 Mar 21;18:223. doi: 10.1186/s13018-023-03687-8 (PMC10031962; doi:10.1186/s13018-023-03687-8)
Supplement: Supplementary file 5 — Additional file 5: Supplement 5 TP, TN, FP, FN data from papers. [file 13018_2023_3687_MOESM5_ESM.pdf]

AUTOR	year	Type	N	tpos	fneg	fpos	tneg	Route
Abele J et al.	2015	General	17	2	0	1	14	i.a
Al-Nabhani et al.	2014	General	24	9	0	3	12	i.v
Arican et al. (a)	2015	Femoral	30	26	4	0	0	i.v
Arican et al. (b)	2015	Tibial	30	28	2	0	0	i.v
Bo Bao et al.	2021	General	36	1	1	1	33	i.a
Chew Ch et al. (a)	2010	Femoral	44	9	3	12	20	i.a
Chew Ch et al. (b)	2010	Tibial	44	6	1	5	32	i.a
Hirshman M et al.	2015	General	33	10	0	1	22	i.v
Mandegaran R et al.	2018	General	41	13	0	6	22	i.v
Murer A et al. (a)	2019	Femoral	83	3	4	0	76	i.v
Murer A et al. (b)	2019	Tibial	83	8	3	0	72	i.v
Murer A et al. (c)	2019	Patelar	83	55	1	2	25	i.v
